# Unveiling the molecular mechanisms of stigmasterol on diabetic retinopathy: BNM framework construction and experimental validation

**DOI:** 10.3389/fmed.2025.1537139

**Published:** 2025-05-09

**Authors:** Hongrong Zhang, Yufan Li, Qi Xu, Zhaohui Fang

**Affiliations:** Anhui University of Chinese Medicine, Hefei, China

**Keywords:** herbal medicine, diabetic retinopathy, machine learning, molecular fingerprint, experimental verification

## Abstract

**Background:**

Diabetic retinopathy (DR), one of the most common complications of diabetes, severely impacts patients’ quality of life. The combined use of the traditional Chinese medicines Astragalus, Fructus ligustris, and Cornus officinalis has yielded considerable therapeutic effects in clinical DR treatment.

**Methods:**

In this study, a multimodule framework (BNM) encompassing bioinformatics, network pharmacology, and machine learning (ML) based on molecular fingerprints was innovatively developed to thoroughly investigate the molecular mechanisms of this Chinese medicine in treating DR.

**Results:**

A total of 40 active components and 12 core targets were identified. Enrichment analysis identified key pathways such as VEGF signaling pathway, TNF signaling pathway and HIF-1 signaling pathway. Prediction models using key targets, such as PPARG, were constructed from the GEO database and validated via immune infiltration analysis and molecular docking, revealing that PPARG may be a potential target for DR treatment. Moreover, the core component of this Chinese medicine, stigmasterol, was identified using a ML model based on molecular fingerprints. *In vivo* experiments demonstrated that stigmasterol can regulate glucose and lipid metabolism, improve systemic inflammatory levels, and ameliorate ocular vascular changes in DR by modulating the expression of PPARG.

**Conclusion:**

The BNM framework suggests that PPARG may be an important target for stigmasterol in the treatment of DR, with its mechanism potentially related to the VEGF/VEGFR pathway.

## 1 Introduction

Diabetic retinopathy (DR) is one of the most common microvascular complications of diabetes. Currently, approximately 103 million people with diabetes worldwide are affected by DR (1). A 2020 study on the causes of blindness and visual impairment revealed that DR is the fifth most common cause of preventable blindness and moderate to severe visual impairment (2). Currently, DR is commonly treated clinically with intravitreal injections of steroids and anti-vascular endothelial growth factor. However, these treatments can have unstable therapeutic effects and considerable side effects (3). Consequently, it is important to find safe and effective new drugs.

Since traditional Chinese medicines (TCMs) are composed of a variety of herbs with extensive active ingredients and drug targets, they have shown outstanding clinical efficacy in the treatment of DR (4). The triplet medicine composed of *Astragalus-Fructus ligustris-Cornus officinalis*, known as HuangQi (HQ)-NvZhenZi (NZZ)-ShanZhuYv (SZY), has been extensively applied in the clinic to treat DR and serves as the core medicinal component in numerous traditional Chinese medicine prescriptions, as it can effectively ameliorate the clinical symptoms of DR (5). According to TCM theory, vision is closely related to the liver, as well as the contents of Qi and blood in humans. HQ supplements Qi whereas NZZ and SZY supplement the blood in liver, which then sends blood upwards to the eyes to supplement Qi and the blood and improve eyesight. Previous laboratory studies have shown that the astragaloside IV contained in HQ inhibits the expression of miR-138-5p, thereby increasing the activities of Sirt1/Nrf2 and the antioxidant capacity of cells to improve ferroptosis and reduce cell death, ultimately inhibiting the progression of DR (6). Luteolin, an effective component of NZZ, can reduce the expression of NLRP1, NOX4, TXNIP, and NLRP3, thereby increasing inflammation and oxidative stress and inhibiting the retinal cell apoptosis in DR rats (7). The quercetin and saponins extracted from SZY can lower blood glucose and blood lipid levels by protecting islet function, thus improving insulin resistance and regulating glucose and lipid metabolism (8–11). Moreover, SZY extract can improve diabetes-related complications (12). However, the specific effects, targets and mechanisms of the combined use of these three drugs have not been systematically studied.

Fortunately, the development of artificial intelligence (AI) technology has brought new opportunities for research in this field. Owing to its excellent data processing capabilities, AI can reveal the deep connections between the chemical components of TCMs, diseases, and targets, providing a new approach to understand the complex interactions between the components of TCMs and elucidating the mechanisms of diseases. For example, Li et al. combined machine learning (ML) and deep learning models to predict antioxidant activity in gentian (13). Gu et al. developed a heterogeneous graph neural network model to predict the compatibility strength and probability among the herbs within the relevant prescriptions of colorectal adenoma (14). Zhang et al. applied the deep learning graph embedding algorithm framework Node2vec, in combination with Danshen and Chuanxiong, to successfully predict new targets for the treatment of cardiovascular diseases (15). These studies indicate that the application of AI technology in TCM research cannot only promote an understanding of the mechanisms of action of TCMs but also provide new perspectives for explaining the interactions between TCMs and diseases.

**FIGURE 1:**
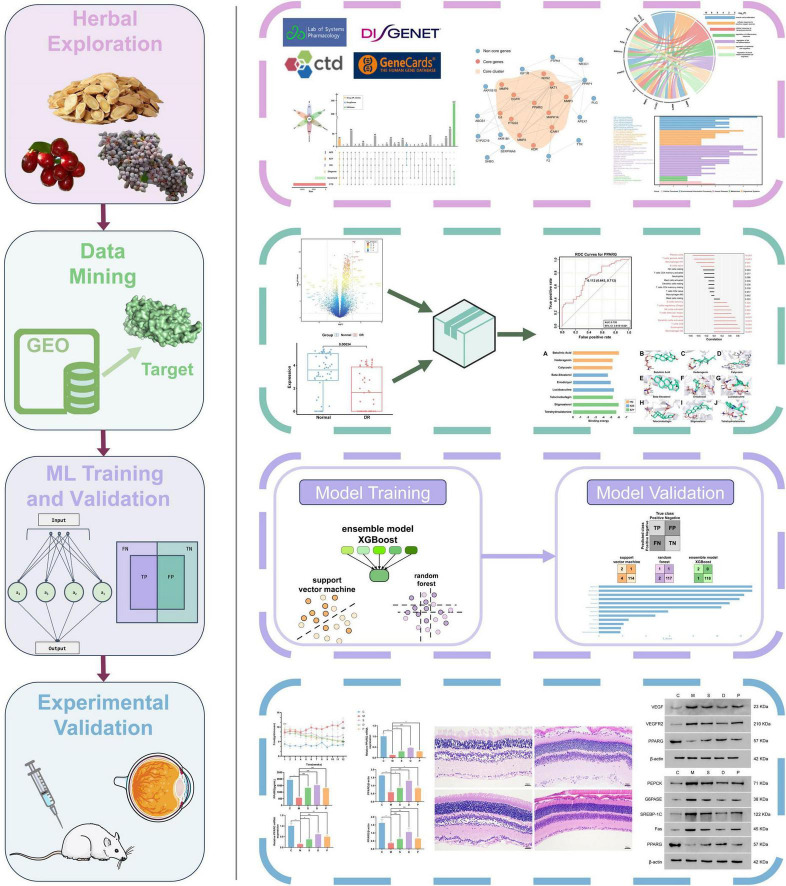
Workflow of this study. Different colors denote different modules. The pink color represents the network pharmacology module, the green color represents the bioinformatics module, the purple color represents the molecular fingerprint-based ML module, and the blue color represents the animal experimental validation module.

In this study, an integrated framework (BNM) encompassing bioinformatics, network pharmacology, and molecular fingerprint ML to assess the potential of the using the triplet medicine HQ, NZZ, and SZY for the treatment of DR was successfully developed and validated. We extracted the active components of these herbs from TCM databases and the literature and performed functional enrichment analysis, constructed a diagnostic model, and carried out immunological analysis and molecular docking by screening core targets and components. Furthermore, we developed a ML model using 990 compounds from the LOPAC-1280 and Prestwick Chemical Library and validated it with the core components of the triplet medicine. We also applied the Breaking of Retrosynthetically Interesting Chemical Substructures (BRICS) and Retrosynthetic Combinatorial Analysis Procedure (RECAP) algorithms for fragment analysis of the core components to evaluate the potential of this model to generate lead components. Finally, these findings were corroborated through animal experiments (Figure 1). In summary, in our research, we evaluated the efficacy of combined DR treatment with the triplet medicine from multiple perspectives, utilized a molecular fingerprint ML model to identify effective TCM components, and provided a novel perspective for drug research and repurposing.

## 2 Materials and methods

### 2.1 Construction of the DR drug-associated gene network

All the components in HQ, NZZ, and SZY were collated on the basis of the Traditional Chinese Medicine Systems Pharmacology Database and Analysis Platform (TCMSP). Generally, oral bioavailability (OB) is one of the most crucial absorption, distribution, metabolism and excretion (ADME) pharmacokinetic parameters, whereas drug likeness (DL) indicates the similarity between the physical properties of the component in question and those of known drugs (16). In this study, the criteria for identifying effective components were set to OB ≥ 30% and DL ≥ 0.18. The relevant targets of the bioactive components were subsequently identified using the SwissTargetPrediction database and standardized using the UniProt database. DR-related genes were obtained from the Comparative Toxicogenomics Database (CTD) and the GeneCards and Disease Gene Network (DisGeNET) databases. The genes associated with diseases and drugs were intersected using the UpSetR package in R software to identify the intersecting genes. Three DR–TCM-related genes were subsequently added to the gene set after review of the literature. A protein–protein interaction (PPI) network was constructed on the basis of the gene sets. The PPI network developed using the STRING platform, an interactive gene database, gave a high confidence interaction score (0.7), indicating a targeted network. All of the database searches were limited the species to *Homo sapiens*.

### 2.2 Core network cluster mining and functional analysis

To further explore the core network, we employed Cytoscape software (version 3.82) to visualize the target network. Network analysis tools were employed to calculate the key network metrics, including the network diameter, the clustering coefficient, and multiedge node pairs. The MCODE plugin, which employs molecular complex detection, was used for core network mining on the target network. The core network group was obtained with the following parameters: degree cut-off = 2, node score cut-off = 0.2, K-core = 2, and max depth = 100.

The clusterProfiler package in R was employed for gene ontology (GO) and Kyoto Encyclopedia of Genes and Genomes (KEGG) analyses of both the target and core networks, and the results were filtered at the level of *p*-value ≤ 0.05. GO analysis encompasses three domains: biological process (BP), molecular function (MF), and cellular component (CC). The GO results were filtered to exclude the terms significantly unrelated to DR. KEGG analysis revealed the top 40 target networks and the top 30 core networks, as the latter results were weakly correlated with DR. The results were plotted using the R packages ComplexHeatmap, ggplot2, and circlize.

### 2.3 Identification of differentially expressed genes (DEGs)

Mining of the core network group and functional analysis revealed that 12 core targets were strongly correlated with DR. To further investigate and confirm the biological functions of the core targets in DR, we randomly selected PPARG for subsequent analysis. The Gene Expression Omnibus (GEO) database includes high-throughput gene expression data, DNA microarray data, and hybridization array data (17). The GSE146615 microarray dataset, including 42 control samples and 52 samples from individuals with DR, was downloaded from the GEO database. Differential expression analysis was performed using the limma package in R. The limma package is renowned for its capabilities in terms of data normalization and precise gene expression data interpretation, and has therefore become the preferred choice for high-throughput and microarray differential analysis (18). The screening criteria were |log (FC)| > 1 and *p*-value < 0.001. The ggplot2 package was employed to generate a volcano plot, which was used to visualize the top 30 DEGs. A focused analysis of PPARG was subsequently conducted to explore its expression in the normal and DR groups, and the data were plotted with the ggpubr package.

### 2.4 Diagnostic prediction model establishment and immune analysis

To evaluate the diagnostic and predictive utility of PPARG for DR, a predictive model was developed using microarray datasets. Analysis was conducted using the pROC package in R software. Binary classification models are commonly evaluated using receiver operating characteristic (ROC) curves (19). The position of the ROC curve in relation to the upper left quadrant is indicative of the classification performance of the method in question. The bootstrap algorithm was employed for statistical analysis. Bootstrap, proposed by Efron, is a simulation-based sampling statistical inference algorithm that does not require assumption of a specific theoretical distribution (20). The area under the curve (AUC) can then be used to assess the predictive accuracy of PPARG. A larger AUC indicates greater accuracy of using PPARG for DR diagnosis. The abscissa False Positive Rate (FPR) and ordinate True Positive Rate (TPR) of the ROC curve can be calculated via the following formula.


(1) $$F\,P\,R=\frac{F\,P}{F\,P+T\,N}$$

(2) $$T\,P\,R=\frac{T\,P}{T\,P+F\,N}$$

Here, TP, TN, FP, and FN represent the numbers of true positive, true negative, false positive, and false negative results, respectively (Equations 1 and 2). TP represents the number of samples correctly predicted as positive by the model. TN represents the number of samples correctly predicted as negative by the model. FP represents the number of samples predicted as positive by the model but that were actually negative. FN represents the number of samples predicted as negative by the model but that were actually positive. FPR is calculated as the proportion of actual negative samples that were incorrectly predicted as positive by the model. TPR is calculated as the proportion of actual positive samples that were correctly predicted as positive by the model.

An investigation was subsequently conducted to determine whether PPARG disrupts normal homeostasis by mediating immune dysregulation, thereby leading to DR. To this end, the CIBERSORT algorithm was employed to assess the correlation between PPARG and 22 immune cell types within the dataset. The CIBERSORT algorithm is a canonical computational method for analyzing immune outcomes. It employs the principles of linear SVM regression to deconvolve the expression profiles of immune cell subtypes and calculate their abundance (21). The statistical threshold was set to *p* < 0.05, and the results were visualized using the ggpubr package in R.

### 2.5 Binding free energy calculations

To evaluate the functional effects of the active components present in HQ, NZZ, and SZY on PPARG, we employed AutoDock software (version 1.5.6) to compute the binding free energies between PPARG and these components. The structure of PPARG was retrieved from the RCSB Protein Data Bank (PDB) database, and the water molecules present were subsequently removed. The structures of the active components of these three medicinal preparations were sourced from the PubChem database. After processing, a Lamarckian genetic algorithm was used to predict the molecular conformations of the protein receptor and small molecules, and the binding energies of the active ingredients were computed separately. These binding energies indicate the affinity of the small molecule for the protein. The binding energies were also calculated via the following formula:


(3) $$\begin{array}{c} \Delta G_{BIND}=\Delta G_{VDW}+\Delta G_{HBOND}+\Delta G_{ELECT}+\Delta G_{TOR}+\\ \Delta G_{DESOLVA} \end{array}$$

In Equation 3, ΔGBIND represents the binding free energy, ΔGVDW denotes the van der Waals potential, ΔGHBOND represents the hydrogen bonding potential, ΔGELECT denotes the electrostatic potential, ΔGTOR represents the dihedral angle torsional energy, and ΔGDESOLVA indicates the free energy of solvation; the units are kcal⋅mol^–1^. The coordinates of the active cavity center of PPARG were defined as x_center = 3.812, y_center = 49.518, and z_center = 67.059, and the search space box was set to 126*126*126. The time of conformational searches was set to 50. ΔGBIND < −5 kcal⋅mol^–1^ indicates good binding capacity, while ΔGBIND < −7 kcal⋅mol^–1^indicates strong binding capacity.

Additionally, the three highest binding energies of the active components with PPARG were selected. The pyplot module in Python was used to construct a bar chart. Finally, the conformations were visualized with PyMOL software (version 2.1) according to the calculated binding free energies.

### 2.6 Drug composition model validation

#### 2.6.1 Data assembly and feature determination

To validate the therapeutic efficacy of the aforementioned nine compounds in DR, a diverse array of compounds was gathered to train a ML model. We collected 394 negative compounds from LOPAC-1280, and defined the 9 components of HQ, NZZ, and SZY as positive compounds. Significantly more negative compounds than positive compounds were included to ensure that the model would be able to identify positive compounds with greater accuracy. Although the majority of the compounds were deemed negative, it is possible that a small proportion of these molecules may have been mislabelled, as there may be undiscovered compounds that could be used to treat DR.

These 403 compounds were converted to SMILES format, and the RDKit package in Python was employed to compute a range of physical and chemical descriptors from 200 unique aspects, including Hybrid Estate-VSA descriptors, topochemical descriptors, and the QED and Lipinski parameters. RDKit is a computational chemistry toolkit that can process molecules, analyze different chemical input formats, filter out unwanted compounds, standardize molecules, and calculate various molecular characteristics (22). The results were plotted using the matplotlib package in Python.

#### 2.6.2 Model training

To increase the computational accuracy and stability of the model, three distinct ML algorithms were employed: XGBoost 2.0.3, random forest (RF), and SVM. Both RF and SVM utilized scikit-learn 1.3.0. The Gini index was used to assess the feature importance of the 200 physicochemical descriptors in the dataset. Feature importance helps to indicate the significance of the physicochemical descriptors, which are believed to be closely linked to classification. When each feature node pertains to the same class, the Gini index is minimal, leading to the highest purity and minimal uncertainty (23).

The acquired feature dataset was subsequently reconstructed and assembled using various models. The dataset containing 403 compounds was split into training and testing sets via stratified random sampling (70% for training and 30% for testing). To ensure fair comparison among the models and robust model performance, the positive samples were fully utilized. Following cross-validation to check for overfitting, model comparisons were assessed via three performance metrics: precision, recall, and F1 score, as follows.


(4) $$P\,r\,e\,c\,i\,s\,i\,o\,n=\frac{T\,P}{T\,P+F\,P}$$

(5) $$R\,e\,c\,a\,l\,l=\frac{T\,P}{T\,P+F\,N}$$

(6) $$F\,1\,s\,c\,o\,r\,e=\frac{2}{\frac{1}{P\,r\,e\,c\,i\,s\,i\,o\,n}+\frac{1}{R\,e\,c\,a\,l\,l}}$$

Precision represents the proportion of samples predicted to be positive by the model that are actually positive, emphasizing the accuracy of the prediction [Equation (4)]. Recall represents the proportion of actual positive samples that were correctly predicted by the model, which emphasizes the coverage of positive samples (Equation 5). F1 score is the harmonic mean of Precision and Recall, which is used to assess the model’s performance in a combined manner (Equation 6). F1 score is designed to balance Precision and Recall, thereby mitigating the extremes that may occur when these metrics are used individually. Precision, recall, and F1 score take values between 0 and 1, with higher values indicating better predictive performance of the model.

Moreover, we considered the possibility of optimizing the three models in accordance with the existing predictions. Encouragingly, the class weight parameters for the SVM and RF models were adjusted to the balanced and default values, respectively. Additionally, the maximum depth of the XGBoost model was 10 and the colsample_bytree was 0.5, which were suggested to be the optimal choices. The optimized model was subsequently retrained, and the results of each prediction were evaluated in detail by plotting the mean AUC, which can eliminate the instances of a single prediction being either poor or overly high, facilitating an objective assessment of the performance of the three models on the dataset (24). Three ML models were compared using the Python package numpy, sklearn, and matplotlib for visualization.

#### 2.6.3 Model evaluation

Upon evaluation, the SVM model exhibited the least predictive power, and the XGBoost model was marginally superior to the RF model in terms of predictive ability. XGBoost was selected for evaluation to ascertain its performance in practical applications, and the dataset was augmented by incorporating 596 compounds from LOPAC-1280 and the Prestwick Chemical Library for a total of 999 distinct compounds. The compounds were subsequently no longer categorized as either negative or positive, enabling the model to autonomously interpret and make decisions on the sole basis of the physical and chemical descriptions, thereby completing the identification task. The data were normalized using XGBoost, and z scores and probabilities were calculated. A z_score greater than 1.83 was considered indicative. The z scores were calculated as follows:


(7) $$\tilde{X}=\frac{1}{n}\,\sum_{i=1}^{n}X_{i}$$

(8) $$Z\,s\,c\,o\,r\,e=\frac{\tilde{X}-E\,\left[X\right]}{\sigma \,X/\sqrt{n}}$$

In Equation (7), $\tilde{X}$ denotes the average value, n represents the number of samples, and *X_i_* represents the observed value of the i-th sample. $\tilde{X}$ is calculated by summing the observed values of all samples and dividing by the number of samples n, thereby reflecting the center of the data set. In Equation (8), E[X] denotes the overall mean. σ*X* denotes the standard deviation. The z_score indicates whether the sample mean deviates significantly from the overall mean, which can serve as one indicator among others for assessing the model’s performance. The results were plotted using the matplotlib package in Python.

#### 2.6.4 Exploring the possibility of identifying lead compounds

Decomposing drug molecules into fragments and constructing chemically feasible fragment libraries are pivotal stages in the drug discovery process. To explore the nine drug components of HQ, NZZ and SZY for lead compound development on the basis of fragments, the RECAP and BRICS algorithms were employed to extract commonalities from the drug molecular fragments. The RECAP algorithm, which is based on retrosynthetic analysis, manipulates a series of cleavage sites to yield a set of plausible drug molecular fragments that adhere to the fundamental principles of practical cleavage tools (25). In contrast, the BRICS algorithm segments molecules on the basis of bond formation (26). Consequently, fragments similar to those generated by BRICS can be used to reassemble new drug molecules, thereby increasing the probability that these fragments can be used to synthesize new drugs (27). Finally, molecular structures were drawn using the rdkit.Chem.Draw package in Python.

### 2.7 Experimental verification in an animal model

#### 2.7.1 Animal grouping, modeling, and treatment

The number of experimental animals per group was calculated using the resource-equation method with the following formula:


(9) E=K⁢(n-1)

In Equation (9), *K* is the number of treatment groups, *n* is the number of animals per group, and *E* is the error degrees of freedom. Setting *E* = 20, the equation yielded *n* = 6 animals per group. To accommodate potential attrition, 10 animals were allocated to each group (28).

Forty 8-week-old SPF male db/db mice and 10 8-week-old SPF male C57BL/6 mice were reared at 22.5°C with 40–50% humidity on a 12/12 h light/dark cycle. The C57BL/6 mice were fed ordinary feed, while the db/db mice were fed a high-fat diet. This study was approved by the Animal Ethics Committee of Anhui University of Chinese Medicine (ethics number: AHUCM-mouse-2024116) and was conducted in strict accordance with the animal experiment guidelines.

After 1 week of adaptive feeding, the db/db mice were randomly divided into a model group (M; 0.2 mL⋅kg^–1^⋅d^–1^ 0.9% NaCl), a positive control group (P; 10 mg⋅kg^–1^⋅d^–1^ pioglitazone + 230 mg⋅kg^–1^⋅d^–1^ calcium oxybenzoate), a Chinese medicine intervention group (D; 910 mg⋅kg^–1^⋅d^–1^), and a single drug intervention group (S; 100 mg⋅kg^–1^⋅d^–1^ stigmasterol), with 10 animals in each group. Once a day for 12 weeks, the C57BL/6 mice in the blank control group (C) were given an equal volume of 0.9% NaCl solution. The general conditions of the mice, such as activity, hair color, and water intake, were observed. Body mass was measured every 3 weeks, and blood glucose level, determined from a sample taken from the tip of the tail, was measured before and 12 weeks after treatment.

#### 2.7.2 Haematoxylin and eosin staining

The retinal tissue of the mice was fixed with 4% paraformaldehyde solution for 24 h, dehydrated, embedded in paraffin and made into 5 μm slices. The slices were treated with xylene, anhydrous ethanol and 75% ethanol in sequence, washed, and stained with haematoxylin for 5 min. Following dehydration with gradient ethanol and staining with eosin solution for 20 min, the pathological morphologies of the retinal tissue slices were observed via optical microscopy after dehydration with anhydrous ethanol and sealing and making transparent with xylene and neutral gum.

#### 2.7.3 Detection of biochemical indices

Fasting blood glucose (FBG), total cholesterol (TC), and triglyceride (TG) levels were assessed using a fully automated biochemical analyser. The levels of glycated hemoglobin A1c (HbA1c), low-density lipoprotein cholesterol (LDL-C), high-density lipoprotein cholesterol (HDL-C), PPARG, tumor necrosis factor-α (TNF-α), interleukin-1β (IL-1β), interleukin-6 (IL-6), and interleukin-10 (IL-10) were determined via enzyme-linked immunosorbent assays (ELISAs).

#### 2.7.4 Western blotting (WB)

The tissue blocks were washed with precooled phosphate-buffered saline (PBS) 2–3 times, cut into small pieces and placed in a homogenization tube, and 10 volumes of lysis solution was added. Homogenization was performed on ice for 30 min with shaking every 5 min to ensure complete tissue lysis. Then, the mixture was centrifuged at 12,000 rpm and 4°C for 10 min, and the supernatant, which contained the total protein, was collected. The protein concentration was determined via the BCA method. After performing gel electrophoresis, transferring to a PVDF membrane, and sealing, the PVDF membrane was immersed in 1:1,000 dilutions of the following monoclonal antibody solutions at 4°C with shaking on a bed overnight: vascular endothelial growth factor (VEGF), VEGFR2, PEPCK, G6PASE, SREGBP-1C, Fas, and PPARG. Then, sheep anti-rat HRP-labeled secondary antibody (diluted 1:5,000) was added for incubation at 37°C for 2 h. The samples were washed with Tris-buffered saline containing Tween-20 (TBST) 3 times for 5 min each time. Finally, the samples were analyzed with an enhanced chemiluminescence (ECL) system.

#### 2.7.5 Quantitative polymerase chain reaction (qPCR)

The tissue blocks were ground completely, and an appropriate amount of TRIzol reagent was added to extract total RNA. Reverse transcription was performed according to the kit instructions, followed by gentle mixing and centrifugation The reverse transcription program was as follows: 25°C for 5 min, 42°C for 30 min, and 85°C for 5 min. After reverse transcription, amplification was performed as follows: predenaturation at 95°C for 5 min; followed by 40 thermal cycles of denaturation at 95°C for 10 s, annealing at 60°C for 20 s, and extension at 72°C for 20 s. β-Actin was selected as the internal reference to determine the Ct value of each target. The relative mRNA expression levels were calculated via the 2-ΔΔCt method. The primers used are shown in Table 1.

**TABLE 1:** Primer sequences used for qPCR.

Gene	Primer	Sequence (5′-3′)
VEGF	F R	TCTTCCAGGAGTACCCCGAC GGGATTTCTTGCGCTTTCGT
VEGFR2	F R	TCCACATGGGCGAATCACTC GCAATTCTGTCACCCAGGGA
PEPCK	F R	TGCGGATCATGACTCGGATG AGGCCCAGTTGTTGACCAAA
G6PASE	F R	GGGCATCAATCTCCTCTGGG GTCCAGGACCCACCAATACG
SREBP-1c	F R	ACTGGACACAGCGGTTTTGA CTCAGGAGAGTTGGCACCTG
Fas	F R	GTGGATCTGGGCTGTCCTG AGCAAAATGGGCCTCCTTGA
PPARG	F R	ATTGAGTGCCGAGTCTGTGG GGCATTGTGAGACATCCCCA
β-actin	F R	GATATCGCTGCGCTGGTCG AGGTGTGGTGCCAGATCTTC

#### 2.7.8 Statistical analysis

The BNM framework was developed using R (version 4.3.0) and Python (version 3.8). Data were analyzed using SPSS version 26.0. The Shapiro-Wilk test was employed to assess the normality of the experimental data. Results are presented as mean ± standard deviation. Homogeneity of variances across groups was evaluated using Bartlett’s test. Differences among groups were analyzed using one-way ANOVA, followed by Tukey’s *post hoc* analysis for multiple comparisons. For comparisons between two groups, an independent samples *t*-test was conducted. A significance level of α = 0.05 was set, and *p* < 0.05 was considered statistically significant. The results of the animal experiments described above were visualized using GraphPad Prism version 9.0.0.

## 3 Results

### 3.1 TCM component mining and target acquisition

**FIGURE 2:**
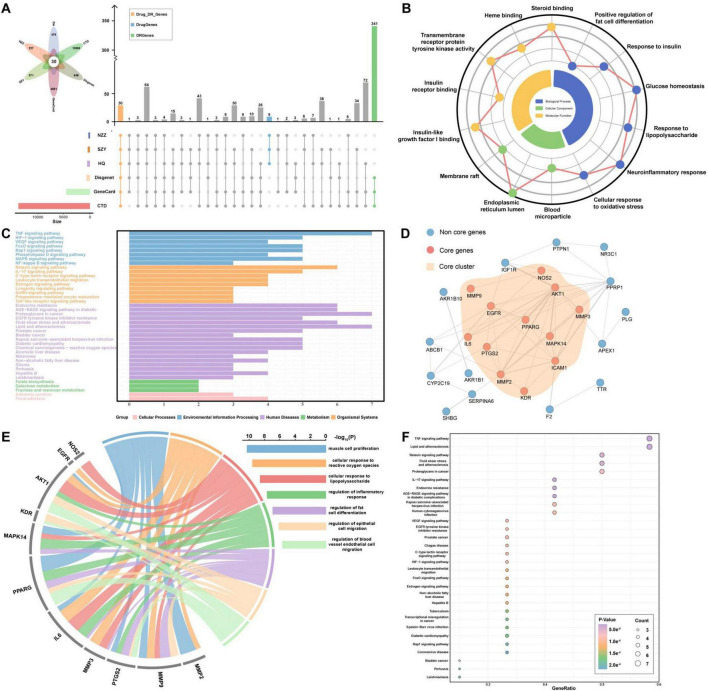
Drug–disease network mining and functional analysis. **(A)** The drug targets of HQ, NZZ, and SZY were intersected with the acquired DR disease targets. **(B)** GO analysis of target network targets; blue represents BPs, yellow represents MFs, and green represents CCs. The closer the dots are to the periphery, the greater the significance of the GO entry. **(C)** KEGG enrichment analysis of the target network. A larger number of genes is associated with a more significant KEGG enrichment result. **(D)** Core network target mining. **(E,F)** GO and KEGG analyses of the core network targets.

TCMSP database screening yielded a total of 40 chemical components, of which 17 were from HQ, 9 were from NZZ, and 14 were from SZY. Mining using Swiss Target Prediction yielded a total of 1071 targets, of which 475 were from HQ, 226 were from NZZ, and 370 were from SZY. A total of 4,560 targets, 648 targets, and 13,901 targets related to DR were subsequently identified from the GeneCards, DisGeNET, and CTD databases, respectively. The UpSetR package was used for intersection of the six different target classes, as shown in Figure 2A, and 30 target intersecting genes were obtained. After literature mining, MAPK14, ICAM1, and IL6 were added to the list intersecting genes to form a gene set. High-confidence interaction scores were subsequently generated using the STRING database to obtain target network clusters, which helped us to better understand the interactions between the therapeutic targets.

### 3.2 Core network construction and functional enrichment analysis

To further explore the interactions in the core network, we analyzed the target network cluster. The target network cluster results were further analyzed using the MCODE plugin in Cytoscape software, and a network cluster consisting of 12 core genes was obtained (Figure 2D), wherein red nodes represent the core network genes and blue nodes represent the noncore network genes. Moreover, we aimed to assess which relevant DR biological processes and pathways might be affected by HQ, NZZ, and SZY. We conducted GO and KEGG analyses on the gene set and the core network cluster. The GO analysis of 33 genes yielded 1,262 entries. After removing the terms clearly unrelated to DR, we found associations with positive regulation of fat cell differentiation, response to insulin, and response to lipopolysaccharide amongst the BPs. The MFs were focused on steroid binding and transmembrane receptor protein tyrosine kinase activity. The CCs were centered on the membrane raft and blood microparticles (Figure 2B). The 12 core network clusters contained a total of 1226 GO entries. Upon comparison, we discovered that the main enriched terms were associated with muscle cell proliferation, the cellular response to reactive oxygen species, and the response to lipopolysaccharide (Figure 2E). Interestingly, both GO analyses included the regulation of fat cell differentiation and the response to lipopolysaccharide, indicating that these two biological processes may be important. Moreover, the KEGG enrichment results for the 33 genes and 12 core network clusters together indicated that the VEGF signaling pathway, TNF signaling pathway and HIF-1 signaling pathway may play key roles in DR therapy, as their degree of enrichment and pathway importance were higher (Figures 2C,F).

### 3.3 Validation of PPARG in clinical samples

**FIGURE 3:**
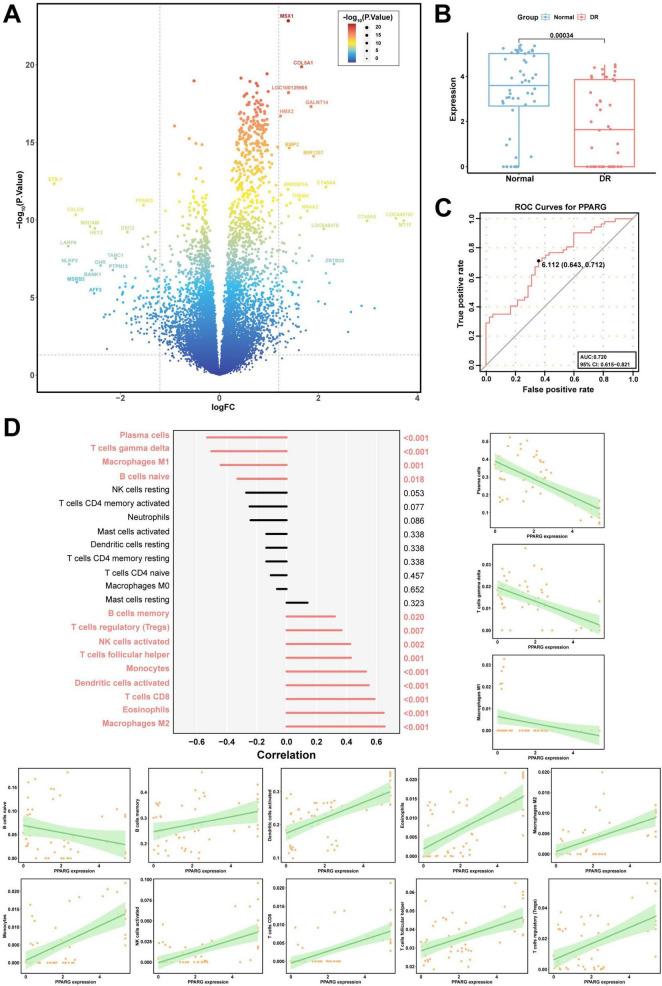
Evaluation of the DEGs, diagnostic modeling, and immunoassays. **(A)** Volcano map of the DEGs from the GSE146615 dataset (Significance increases from blue to red, with deeper red indicating higher significance of DEGs). **(B)** Differential expression of PPARG in DR and normal tissues in the GSE146615 dataset. **(C)** ROC curve and AUC of PPARG for DR diagnosis. The ROC curve is depicted as a red line. **(D)** PPARG and immune cells: plasma cells, gamma delta T cells, M1 macrophages, naive B cells, M2 macrophages, eosinophils, CD8 T cells, activated dendritic cells, monocytes, follicular helper T cells, activated NK cells, regulatory T cells (Tregs), and memory B cells. The green shading represents the confidence interval of the fitted curve.

To explore the functions of the core network genes in DR, we selected PPARG from the GSE146615 microarray dataset as the target for further investigation. After filtering and screening, 331 genes were differentially expressed, amongst which 162 were upregulated and 169 were downregulated. The volcano plot displaying the top 30 DEGs (Figure 3A) revealed a significant difference in the expression of PPARG, which was consistent with our previous analysis. Additionally, after performing a separate analysis of the PPARG gene, we found that the expression of PPARG in the DR group was very significantly lower than that in normal tissue (*p* < 0.001) (Figure 3B).

### 3.4 Analysis of the diagnostic prediction model performance and immune microenvironment analysis

To evaluate the diagnostic value of PPARG for DR, we established a PPARG diagnostic prediction model on the basis of the GSE146615 dataset. The TPR and FPR of PPARG were evaluated via ROC curve analysis, and the results revealed that the AUC value of PPARG was 0.720 (95% CI: 0.615–0.821) (Figure 3C). These findings indicate that PPARG has high diagnostic value for DR. To further explore whether PPARG affects the development of DR through immune cells, we evaluated the relationship between PPARG expression and immune cells in DR using the CIBERSORT algorithm. Considering *p* < 0.05 as a correlation, PPARG was negatively correlated with four immune cells (Figure 3D), namely, plasma cells (*p* < 0.001), gamma delta T cells (*p* < 0.001), M1 macrophages (*p* = 0.001) and naive B cells (*p* = 0.018). PPARG was positively correlated with the infiltration of 9 types of immune cells, namely, M2 macrophages (*p* < 0.001), eosinophils (*p* < 0.001), CD8 T cells (*p* < 0. 001), activated dendritic cells (*p* < 0.001), monocytes (*p* < 0.001), follicular helper T cells (*p* = 0.001), activated NK cells (*p* = 0.002), regulatory T cells (Tregs) (*p* = 0.007) and memory B cells (*p* = 0.02). Taken together, these findings suggest that PPARG may play a key role in the progression of DR by regulating immune cells in the body.

### 3.5 Analysis of the binding free energies

**FIGURE 4:**
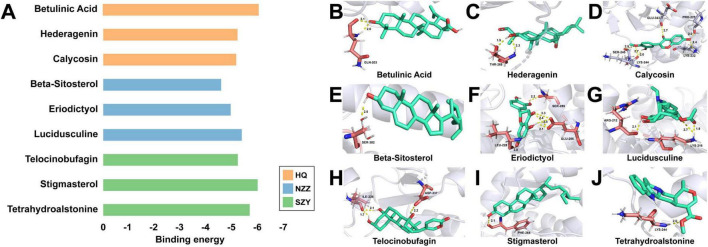
Binding free energy calculations. **(A)** Binding free energies of nine herbal components to PPARG. **(B–J)** Betulinic acid, hederagenin, calycosin, β-sitosterol, eriodictyol, lucidusculine, telocinobufagin, stigmasterol, and tetrahydroalstonine bind to PPARG with different binding modes. The green compound structure represents the small-molecule drug, the white indicates the protein, and the red highlights distinct protein residues.

To identify the ability of the identified components to bind to the target, we calculated the binding free energies between PPARG and selected components. The structures of 40 components were obtained from PubChem, of which 17 were from HQ, 9 were from NZZ and 14 were from SZY. The protein structure of PPARG was obtained from the RCSB PDB database (ID: 8aty). The 40 drugs were docked to PPARG, and the three drugs with the highest binding free energies were selected for visualization (Figure 4A). The results indicated that the three most active components of HQ and their binding free energies with PPARG were −6.01 kcal⋅mol^−1^ (betulinic acid), −5.17 kcal⋅mol^–1^ (hederagenin), and −5.12 kcal⋅mol^–1^ (calycosin). The three most active components of NZZ and their binding free energies with PPARG were −4.54 kcal⋅mol^–1^ (β-sitosterol), −4.9 kcal⋅mol^–1^ (eriodictyol), and −5.33 kcal⋅mol^–1^ (lucidusculine). Finally, the three most active components of SZY and their binding energies with PPARG were −5.18 kcal⋅mol^–1^ (telocinobufagin), −5.94 kcal⋅mol^–1^ (stigmasterol), and −5.64 kcal⋅mol^–1^ (tetrahydroalstonine). Figures 4B–J show that the components adopted different binding modes at the PPARG site, forming hydrogen bonding, H-π bonding and π-π bonding interactions. The above results indicate that these components have good, stable binding to PPARG in terms of binding free energy, verifying the therapeutic effects of HQ, NZZ, and SZY in DR.

### 3.6 Assessment of feature importance in the ML models

**FIGURE 5:**
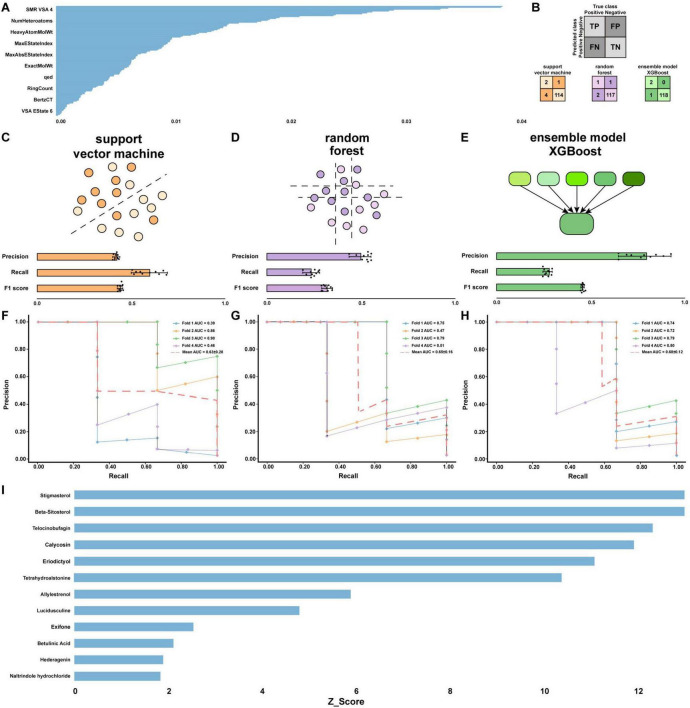
Drug composition ML data feature screening and model training, testing and evaluation. **(A)** Feature importance of 200 physicochemical descriptors in the data model using the RDKIT ML model. **(B)** Three ML algorithm models, SVM, RF and XGBoost, were evaluated on the test set to obtain confusion matrices. **(C–E)** The SVM, RF, and XGBoost algorithms were trained for cross-validation to compute three metrics: precision, recall and F1 score. The bar graph represents the average performance, and the error bars represent the standard deviation. **(F–H)** The SVM, RF and XGBoost algorithms were cross-validated 4 times to obtain precision-recall results and mean precision-recall values. **(I)** A dataset of 999 unclassified compounds was subsequently evaluated using the XGBoost ML algorithm and assigned a z score to indicate their potential as a drug to treat DR, with a higher z score representing a greater probability of treating DR.

We initially assembled a dataset of 9 DR (positive) and nonDR (negative) compounds identified via TCM mining. The nonDR data include a wide range of FDA-approved or clinical-stage compounds from LOPAC-1280, and 394 compounds were identified as clearly not associated with DR, such as adaphostin, trimipramine maleate, and propionylpromazine hydrochloride, upon analysis of both the LOPAC-1280 database and literature mining. The chemical structures of the compounds in the dataset were then converted into numerical format for model training, with 0 (negative) and 1 (positive), and 200 physicochemical descriptors were calculated using the RDKit package. We subsequently used RF to determine the feature values for model validation on the basis of physicochemical descriptors, performed feature selection on the entire dataset, and filtered out feature-irrelevant physicochemical descriptors to intervene in the impurity metric via the Gini index. After normalization, we obtained 120 feature values (Figure 5A and Supplementary Table 1), which were used in conjunction with a dataset of 403 compounds to train the different models for DR analysis.

### 3.7 Model validation

We then attempted to train the dataset using three different ML models: SVM, RF and XGBoost. Although the SVM algorithm is usually applied to linearly divisible datasets, the clever feature of SVM is that it can use kernels for classification and is therefore equally balanced when the data are not divisible. The dataset was randomly divided into a training set (split = 0.7) and a test set, with the training set containing 282 samples and the test set containing the remaining 121 samples. A fair comparison of performance between the models was made on the basis of cross-validation of the datasets and full use of the positive samples. After testing (Figure 5B), we found that the TN, TP, FN, and FP values were 114, 2,4 and 1 for the SVM model; 117, 1, 2, and 1 for the RF model; and 118, 2, 1, and 0 for the XGBoost model, respectively. We also used three performance metrics, precision, recall and F1 score, to evaluate the performance of the three models (Figures 5C–E). We believe that false positives are more detrimental than false negatives in early drug development, as they can lead to the mistaken assumption that compounds are effective, thereby increasing the cost of downstream drug development.

We also considered that the precision, recall and F1 scores could be effectively improved by adjusting the SVM and RF class weights as well as increasing the maximum depth of XGBoost. Through cross-validation, we found that the mean AUCs for the SVM, RF and XGBoost models were 0.63 ± 0.28, 0.65 ± 0.16, and 0.68 ± 0.12, respectively (Figures 5F–H). On the basis of the mean AUCs and considering the heterogeneity of the data, we believe that all three models have excellent performance.

### 3.8 Model prediction

Although all three models all had excellent performance, we focus mainly on the XGBoost model because of its lack of false positives and higher mean AUC, indicating higher accuracy. After adding the compounds from LOPAC-1280 and the Prestwick Chemical Library, we composed a dataset containing a more compounds (999). The abilities of these compounds to treat DR were predicted on the basis of their physicochemical characteristics using the XGBoost model. Figure 5I shows that most of the nine compounds derived from herbs had high z scores, among which stigmasterol ranked the highest, indicating that stigmasterol might be the best drug from HQ, NZZ, and SZY for DR treatment. Interestingly, three compounds, allylestrenol, exifone, and naltrindole hydrochloride, had higher z scores than some of the other compounds. Nevertheless, we believe that this finding is reasonable because there may be some compounds with undiscovered therapeutic effects on DR.

### 3.9 Fragmentation analysis of nine components

**FIGURE 6:**
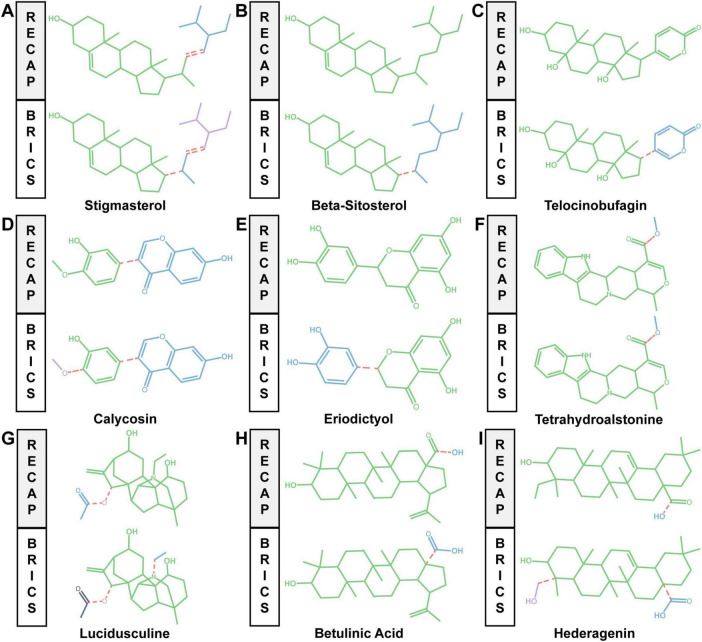
Generation of drug molecular fragments. **(A–I)** Molecular fragments of stigmasterol, β-sitosterol, telocinobufagin, calycosin, eriodictyol, tetrahydroalstonine, lucidusculine, betulinic acid, and hederagenin obtained using the RECAP and BRICS algorithms. Different molecular fragments are represented using distinct color codes, with red dotted lines indicating the broken bonds.

To explore the contributions of the functional groups of the nine components in the triplet medicine and assess whether these components can be modified to become lead components, we performed fragment segmentation of nine components using the RECAP and the BRICS algorithms. Fragment-based drug discovery (FBDD) is a common lead discovery approach that differs from high-throughput screening (HTS) in that early FBDD results may have more applicable physicochemical properties. Upon segmentation by both algorithms, we found that the BRICS algorithm, which is based on bond synthesis, separated β-sitosterol, telocinobufagin, eriodictyol, tetrahydroalstonine, and betulinic acid into two fragments each; stigmasterol, calycosin, and hederagenin into three fragments each; and lucidusculine into four fragments. However, the RECAP algorithm, which differs in that it is based on retrosynthesis, split stigmasterol, calycosin, tetrahydroalstonine, betulinic acid, and hederagenin into 2 fragments each; lucidusculine into 3 fragments; and was unable to fragment β-sitosterol, telocinobufagin or eriodictyol. Interestingly, the segmentation results for tetrahydroalstonine were consistent for both algorithms (Figures 6A–I). In conclusion, these nine components can be further developed into lead components and thus new drugs on the basis of their fragments through the contributions of their respective functional groups.

### 3.10 The effects of drug intervention on the general condition of DR model mice

**FIGURE 7:**
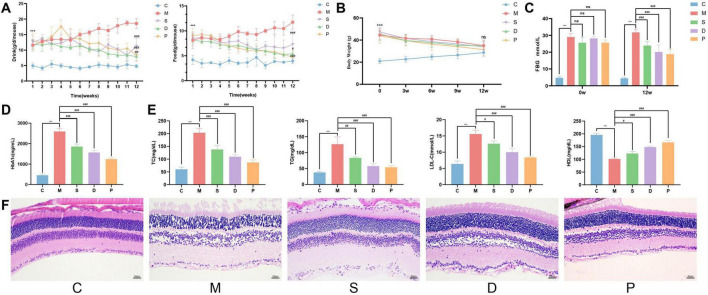
The general condition of the mice in each group. **(A)** Consumption of water (left) and food (right). **(B)** Changes in body weight over 12 weeks. **(C)** FBG levels at 0 w and 12 w. **(D)** HbA1c levels at 12 w. **(E)** The levels of lipid-related indices (from left to right: TC, TGs, LDL-C, and HDL-C). **(F)** HE-stained images of retinal tissue. Compared with the C group, *p < 0.05, **p < 0.01, ***p < 0.001. Compared with the M group, ^#^*p* < 0.05, ^##^*p* < 0.01, ^###^*p* < 0.001. ns, no significance.

Before drug intervention, the mice in group C had neat and shiny fur, were responsive, were agile, and had normal food and water intake as well as body weight. Compared with those in group C, the mice in groups M, D, S, and P had greasy fur, were less responsive, and presented distinct symptoms of diabetes, with increased food and water intake, a significant increase in body weight (*p* < 0.05) (Figures 7A,B), and a marked increase in FBG levels (*p* < 0.05) (Figure 7C). After 12 weeks of drug intervention, compared with the mice in group M, the greasiness of the fur in groups D, S, and P improved, their responsiveness gradually increased, food and water intake decreased (p < 0.05), and body weight gradually decreased, although there was no significant difference compared with that in the group M (Figures 7A,B). Compared with group C mice, group M mice had significantly greater FBG, HbA1c, TC, TG, and LDL-C levels after 12 weeks of intervention (*p* < 0.05) and a significantly lower HDL-C level (*p* < 0.05). Compared with the group M mice, the mice in groups D, S, and P had significantly lower FBG, HbA1c, TC, TG, and LDL-C levels (*p* < 0.05) and a significantly higher HDL-C level (*p* < 0.05) (Figures 7C–E).

### 3.11 The effect of drug intervention on the pathological morphology of the retina in DR model mice

In group C mice, the retinal structure was clear and intact, had neatly arranged cells in each distinct layer, and a complete and continuous inner retinal membrane. Compared with the mice in group C, the mice in group M had obvious vascular dilatation of the retina, visible neovascularisation, unclear hierarchies, loose and irregularly arranged cells in each layer, an obvious reduction in the number of cells, vacuole-like changes in some intercellular spaces, swelling of the inner retinal borders, uneven surfaces, and the local detachment of retinal ganglion cells (RGCs). Compared with the mice in group M, the mice in groups D, S, and P showed different degrees of improvement in retinal morphology, with reduced vasodilatation, less neovascularisation, clear layers, significantly more cells in all layers that were more neatly arranged, fewer vacuole-like changes, and less RGC detachment. Among the groups, groups D and P showed more obvious improvements in DR symptoms and more normal retinal morphologies (Figure 7F).

### 3.12 Effects of drug intervention on the expression of PPARG and inflammatory factors in DR model mice

**FIGURE 8:**
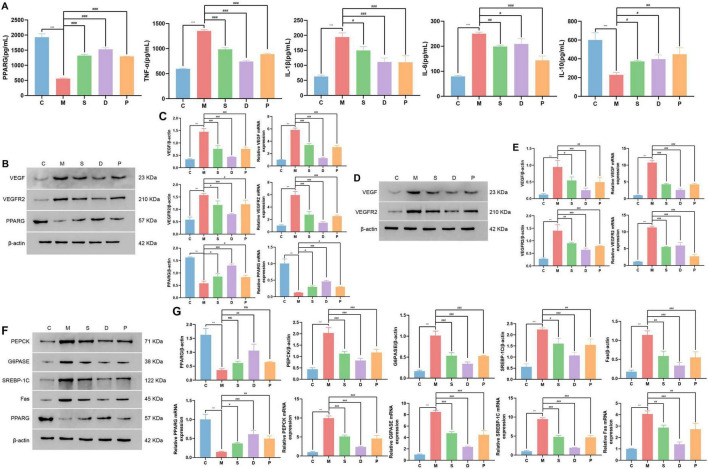
Expression of related factors in the liver and retina of each group of mice. **(A)** Detection of serum PPARG and inflammatory factors via ELISA (from left to right: PPARG, TNF-α, IL-1β, IL-6, and IL-10). **(B,C)** Results of WB and qPCR analysis of VEGF, VEGFR2, and PPARG in the retina. **(D,E)** Results of WB and qPCR analysis of VEGF and VEGFR2 in the liver. **(F,G)** Liver glucolipid metabolism-related indices determined via WB and qPCR (from left to right: PPARG, PEPCK, G6PASE, SREBP-1C and Fas). Compared with the C group, *p < 0.05, **p < 0.01, ***p < 0.001. Compared with the M group, ^#^*p* < 0.05, ^##^*p* < 0.01, ^###^*p* < 0.001. ns, no significance.

Determination of the levels of inflammatory factors in different groups of mice via ELISA revealed that serum TNF-α, IL-1β, and IL-6 levels were significantly greater in group M mice than in group C mice (*p* < 0.05). Compared with those of the mice in group M, the serum TNF-α, IL-1β, and IL-6 levels of the mice in groups D, S, and P significantly decreased to different degrees (*p* < 0.05) (Figure 8A). Since PPARG is an important inflammatory regulator (29), we detected the protein and mRNA levels of PPARG in retinal tissues via WB and qPCR and in serum via ELISA and found that the intraretinal expression of PPARG was lower in the mice in group M than in the mice in group C. Moreover, compared with that in the mice in group M, the intraretinal expression levels of PPARG in the mice in groups D, S, and P were significantly elevated (Figures 8A–C).

### 3.13 Effects of drug intervention on the expression of VEGF and VEGFR2 in the retinal tissue of DR model mice

Angiogenesis is the main pathogenic mechanism of DR, and VEGF is an important marker of angiogenesis. Upon detection of VEGF and VEGFR2 expression in the retinas of the experimental mice via WB and qPCR, we found that VEGF and VEGFR2 were upregulated in the retinas of the mice in group M compared with those of the mice in group C. Compared with those of the mice in group M, the expression levels of VEGF and VEGFR2 in the retinas of the mice in groups D, S, and P were reduced to different degrees (Figures 8B,C).

### 3.14 Effects of drug intervention on the expression of PPARG and markers of glucose and lipid metabolism in the livers of DR model mice

Since PPARG plays important roles in fatty acid storage and glucose metabolism, on the basis of its upregulation in the retina, we examined the levels of PPARG and glycolipid metabolism-related indices in the liver. The results revealed that PPARG was downregulated in the livers of group M mice compared with those of group C mice and was upregulated in the livers of group D, S, and P mice compared with those of group M mice (Figures 8F,G). The expression levels of PEPCK, G6PASE, SREBP-1c, and Fas were increased in the livers of the mice in group M compared with those in group C but decreased to varying degrees in the livers of the mice in groups D, S, and P compared with those in group M (Figures 8F,G). Similarly, we examined VEGF and VEGFR2 expression in the liver, and the results revealed that VEGF and VEGFR2 were upregulated in the livers of the mice in group M compared with that of the mice in group C and downregulated in the livers of the mice in groups D, S, and P compared with that of the mice in group M (Figures 8D,E).

## 4 Discussion

DR is a common microvascular complication of diabetes caused by sustained hyperglycaemia that leads to tissue, nerve, and microcirculation disorders in the eye, ultimately resulting in damage to or the loss of visual function. HQ, NZZ, and SZY are commonly used Chinese herbs for treating DR. Together, these herbs nourish liver blood to brighten the eyes and have shown significant therapeutic effects in the clinical treatment of DR. The complexity of the diverse components and vast data have led to significant limitations in elucidating the material basis of Chinese herbal components and their specific mechanisms of action in disease treatment. AI technology, with its powerful data processing and pattern recognition capabilities, has shown great potential in analyzing the complex chemical component TCM datasets. In this study, we applied AI technology to explore the therapeutic mechanisms of the herbal trio HQ–NZZ–SZY in detail, aiming to provide more substantial scientific evidence for clinical application.

In this study, the core components of HQ, NZZ, and SZY were mined on the basis of OB ≥ 30% and DL ≥ 0.18. A comprehensive analysis of their predicted target genes was also conducted. The targets of the TCM were merged with genes related to DR from the CTD, GeneCards, and DisGeNET databases to construct a core target network, which led to the identification of 12 core genes, including PPARG (Figure 2D). The differential expression of PPARG in DR and its significant diagnostic and predictive capabilities were validated on public databases (Figures 3A–C). Animal experiments confirmed that the level of PPARG in the retinal tissue of DR model mice was significantly lower than that in the mice in the blank control group (Figures 8B,C). The liver is an important site for glucose and lipid metabolism and can store glucose by synthesizing glycogen and regenerate glucose via gluconeogenesis under fasting conditions. Therefore, the expression of PPARG in the livers of DR model mice was evaluated and it was revealed that, compared with that in the blank control group, the expression of PPARG in the livers of DR model mice also tended to decrease (Figures 8F,G). Some other core genes also play important roles in the development and progression of DR. Studies have shown that increased MAPK14 levels inhibit IGFBP-3 expression, and intravitreal injection of IGFBP-3 can reduce TNF-α levels in the retinal tissue of diabetic rats, thereby potentially mitigating retinal damage caused by inflammatory responses. Elevated proinflammatory factors increase ICAM1 expression, which contributes significantly to microcirculation disorders in diabetic patients, leading to leukocyte adhesion and aggregation in retinal vessel walls, which disrupts the blood-retina barrier and damages retinal nerve cells (33–35).

To study the potential therapeutic effects of the TCM herbal trio HQ, NZZ and SZY on DR, the interactions of their main components with PPARG were explored via molecular docking. It was revealed that the nine components betulinic acid, hederagenin, calycosin, β-sitosterol, eriodictyol, lucidusculine, telocinobufagin, stigmasterol, and tetrahydroalstonine strongly bind to PPARG (Figures 4A–J). This implies that this herbal trio may play an important role in the treatment of DR by regulating the expression levels of PPARG. To further explore the therapeutic effects of these nine components on DR, three ML models integrating molecular fingerprint techniques, SVM, RF, and XGBoost, were constructed for their systematic evaluation. Among the evaluated models, the XGBoost model achieved the highest mean AUC and showed better performance in terms of precision, recall, and F1 score, along with improved confusion matrix metrics (TP, TN, FP, and FN), compared with the other two models (Figures 5A–H). Therefore, further data mining was performed using the XGBoost model. The results showed that stigmasterol had the greatest z score, suggesting that stigmasterol may exhibit significant efficacy in the treatment of DR (Figure 5I).

FBDD plays a significant role in drug discovery and development. Structure-based fragmentation is a methodology used in drug design and compound synthesis to decompose compounds into small molecular fragments, thereby identifying and optimizing key structural features and conformational relationships (36). The algorithms used for fragmentation analysis in this study are the BRICS algorithm and the RECAP algorithm. Both algorithms are mainly based on the principle of trans-synthesis, where a set of rules controls the cleavage of chemical bonds, usually emphasizing the selective cleavage of acyclic bonds (37). It was found that stigmasterol yielded different fragmentation patterns when analyzed with the two algorithms; specifically, the BRICS algorithm identified an additional set of carbon chain breaks compared to the RECAP algorithm, suggesting that it may be necessary to evaluate the chemical units synthesized (Figure 6A). The remaining eight drug components are also somewhat amenable to molecular fragmentation segmentation using algorithms, suggesting that they also have potential as lead components (Figures 6B–I).

Stigmasterol is a plant-derived sterol widely present in various plants and foods, such as vegetable oils, nuts, and grains, that has a tricyclic chemical structure similar to that of cholesterol (38, 39). Stigmasterol also has good anti-inflammatory, immunomodulatory, antioxidant, and hypoglycaemic effects (40). Studies have shown that stigmasterol can improve insulin deficiency in patients with diabetes by inhibiting pancreatic cell apoptosis and, in some studies, exhibits activity as an α-glucosidase inhibitor, which is considered to play an important role in lowering blood sugar (41, 42). Furthermore, the efficacy of the TCM herbal trio and stigmasterol were validated in a DR mouse model. Compared with those of the control group mice, the retinal cells of the model group mice presented unclear boundaries, dilated blood vessels, newly formed blood vessels (Figure 7F). Moreover, the FBG, HbA1c, TC, TG, and LDL-C levels significantly increased (*p* < 0.001), whereas the level of HDL-C decreased (*p* < 0.001). Intervention with the TCM herbal trio or stigmasterol significantly reduced the FBG, HbA1c, TC, TG, and LDL-C levels (*p* < 0.05) while increasing the HDL-C level (*p* < 0.05) (Figures 7C–E). Furthermore, in the retinal tissue, blood vessel dilation was alleviated, there were fewer new blood vessels, the cell layers were distinguishable, there were significantly more cells in each layer, the arrangement was more orderly, vacuolar changes were diminished, and there was less shedding of RGCs, collecting approaching normal retinal morphology (Figure 7F). Previous experimental evidence has suggest that stigmasterol can enhance the expression levels of PPARG *in vivo* (43). In the course of our research, intervention with the TCM herbal trio or stigmasterol increased the expression of PPARG (*p* < 0.05) (Table 2), indicating that both the herbal trio and stigmasterol may play a role in lowering blood sugar, regulating lipids, and improving ocular fundus lesions in DR by regulating the expression of PPARG.

**TABLE 2:** Research comparison.

Target	Our research	Previous research
PPARG	BNM: Computational analysis shows that PPARG exhibits higher expression levels in the normal group and lower expression levels in the DR group. Experiment: The intraretinal expression of PPARG was lower in mice of group M compared to those in group C, and was significantly elevated in groups D, S, and P.	In STZ-induced SD rat models, PPARG expression is significantly downregulated, while inflammatory factors IL-1β, IL-6, and TNF-α are markedly upregulated (30).
IL-6	BNM: Computational analysis suggests that IL-6 may be a core gene. Experiment: Serum IL-6 levels were significantly higher in group M mice than in group C mice and were significantly reduced in groups D, S, and P compared with those in group M.	The researchers discovered that, in LM rats, retinal damage became evident at 30 weeks, which coincided with a significant elevation in serum IL-6 expression levels (31).
VEGF and VEGFR2	BNM: Computational analysis identifies VEGF signaling pathway as a potential core regulatory pathway. Experiment: VEGF and VEGFR2 were upregulated in the retinas of mice in group M compared with those in group C, and the expression levels of VEGF and VEGFR2 in the retinas of mice in groups D, S, and P were reduced to varying degrees.	The expression levels of VEGF and VEGFR2 were significantly upregulated in the retinal tissue of STZ-induced Lewis mice, whereas anti-VEGF treatment markedly downregulated their expression levels (32).

Moreover, PPARG is an important regulatory factor in glucose and lipid metabolism that can improve insulin resistance and enhance the sensitivity of target organs to insulin; thus, PPARG has a role in lowering blood sugar (44–48). PEPCK, G6Pase, SREBP-1c and Fas are important indicators reflecting the status of glycolipid metabolism in the liver: PEPCK and G6Pase are the rate-limiting enzymes in glycogenolysis and gluconeogenesis in the liver (49). SREBP-1c regulates the conversion of glucose to lipids in the liver. Inhibiting SREBP-1c expression can effectively reduce fat production and lipid accumulation (50, 51). Fas is a key enzyme in the conversion of glucose to fatty acids (52–54). Fas inhibitors can effectively increase insulin sensitivity and play an important role in maintaining glucose homeostasis in the body (55). Therefore, in this study, the levels of the abovementioned glucose and lipid metabolism-related indicators in the liver were evaluated. Compared with those in group C, the expression levels of PEPCK, G6Pase, SREBP-1c, and Fas were increased in the livers in group M (*p* < 0.001). Furthermore, compared with those in group M, the expression levels of PEPCK, G6Pase, SREBP-1c, and Fas in the livers in groups D, S, and P decreased to varying degrees (*p* < 0.05) (Figures 8F,G). A cohort study shows that improved glucose and lipid metabolism and controlling blood sugar and lipid levels in T2DM can reduce microvascular complications like DR (56). These findings indicate that the TCM herbal trio and stigmasterol can regulate glucose and lipid metabolism possibly through promoting the activity of PPARG, thereby playing a therapeutic role in DR.

On the other hand, PPARG is an important immunomodulator that is closely associated with the expression levels of inflammatory mediators and the inflammasome activation (57). Analysis of the interactions between PPARG and the immunological microenvironment revealed significant correlations between PPARG and 13 different types of immune cells. Specifically, PPARG is negatively correlated with plasma cells, gamma delta T cells, M1 macrophages, and naive B cells, which may suggest that these cells are more active in environments with low PPARG expression or are related to the suppressive effects of PPARG. Conversely, PPARG is positively correlated with nine types of immune cells, including M2 macrophages, which may indicate that these cells play a positive role in microenvironments where PPARG expression is active (Figure 3D). Persistent hyperglycaemia can lead to chronic inflammation within the body, and as the inflammatory response intensifies, inflammatory cells infiltrate and damage retinal tissues, exacerbating retinal vascular permeability, vascular dilation, and retinal thickening (58). The upregulation of PPARG cannot only redirect sugar metabolism but also alleviate the inflammatory response within the body, thereby reducing the damage caused by hyperglycaemia (30). Studies have shown that stigmasterol reduces the expression levels of serum IL-1β, IL-6, TNF-α, and other inflammatory mediators, which may be due to its ability to activate PPARG. This activation can improve the balance between Treg and Th cells and reduce systemic inflammation (59). Moreover, in the serum of the DR model mice, it was found that compared with those in group C, the levels of the proinflammatory factors TNF-α, IL-1β, and IL-6 were significantly increased (*p* < 0.001) and the level of the anti-inflammatory factor IL-10 was decreased (*p* < 0.001) in group M (Table 2). Additionally, compared with group M, the levels of TNF-α, IL-1β and IL-6 were reduced (*p* < 0.05) while IL-10 was increased (*p* < 0.05) in the groups D, S, and P (Figure 8A). These findings indicate that the TCM herbal trio and stigmasterol can reduce the levels of proinflammatory factors and increase the levels of anti-inflammatory factors in the body, thereby improving inflammation in DR.

In this study, in-depth GO and KEGG enrichment analyses of the 12 core targets of the TCM herbal trio were conducted. The analyses revealed that three signaling pathways, including the VEGF signaling pathway, were significantly enriched (Figure 2F). VEGF is an important factor that regulates angiogenesis. In DR, VEGF can bind to its receptor (VEGFR2), which promotes the formation of new blood vessels at the fundus and increases vascular permeability. Compared with normal blood vessels, these pathological new blood vessels lack stable endothelial cells and peripheral supporting cells, making them structurally immature and prone to rupture, which can cause damage to and bleeding in the retina and lead to manifestations such as cystoid macular oedema and optic disc oedema, which are characteristic of DR (32, 58). Studies have shown that increased VEGF expression can activate PPARG phosphorylation, inducing lipid synthesis that promotes T-cell activation, thereby maintaining the activated state of T cells and promoting the occurrence of inflammatory responses (60). Therefore, changes in the expression of the factors in VEGF pathway may initiate the induction of fundus lesions in DR. Consequently, the expression of VEGF and VEGFR2 in retinal and liver tissues was detected. Compared with those in group C, the expression levels of VEGF and VEGFR2 in the retina and liver in group M were increased (*p* < 0.001) (Table 2), but compared with those in group M, the expression levels of VEGF and VEGFR2 in the retina and liver in groups D, S, and P decreased (*p* < 0.05) (Figures 8B–E), indicating that the VEGF/VEGFR2 pathway may be the mechanism by which the TCM herbal trio and stigmasterol exert their therapeutic effects on vascular lesions in the fundus of DR patients.

In summary, in this study, a multimodule framework (BNM) that integrates bioinformatics, network pharmacology, and ML based on molecular fingerprints was developed to thoroughly investigate the potential mechanisms of action of the TCM formula consisting of HQ, NZZ, and SZY on DR, and the mechanisms were validated with animal experiments. This study elucidates the specific molecular mechanisms by which these TCM components treat DR from both theoretical computational and biological experimental perspectives, offering a new perspective for medical research. *In vivo* experiments have demonstrated that the combined HQ-NZZ-SZY formula, along with their key component stigmasterol, can modulate the expression of PPARG. This modulation helps regulate glucose and lipid metabolism while reducing systemic inflammation, thereby inhibiting retinal neovascularization and attenuating the progression of DR. Given that insulin resistance constitutes a critical pathophysiological mechanism in diabetes, based on the aforementioned experimental findings, we tentatively propose that stigmasterol may serve as a potential PPARG modulator for diabetic patients exhibiting significant insulin resistance. This could not only delay DR progression but also ameliorate hyperglycemia and dyslipidemia induced by insulin resistance. However, this study still has some limitations. First, although the molecular mechanism of DR regulation by stigmasterol in this study was investigated by the multimodal BNM framework and verified by animal experiments, the computational results may be biased due to the limited generalization ability of machine learning algorithms and the complexity and diversity of biological systems, and further experiments with human samples are still needed to verify our findings. Secondly, the FBDD-based drug research design in this study is still at a preliminary theoretical stage, necessitating further investigations. Finally, this study shows that upregulation of PPARG inhibits the VEGF/VEGFR2 pathway, thereby attenuating key pathological features of DR. Future in-depth studies are planned to elucidate the precise underlying molecular mechanisms. Therefore, future work will focus on collecting more clinical trial data and animal experimental models for more in-depth research.

## Data Availability

Publicly available datasets were analyzed in this study. The TCM data were retrieved from TCMSP and GSE146615 was retrieved from the GEO database. Further inquiries can be directed to the corresponding authors.
