# Supplementary material for: Unveiling the molecular mechanisms of stigmasterol on diabetic retinopathy: BNM framework construction and experimental validation
Source: Front Med (Lausanne). 2025 May 9;12:1537139. doi: 10.3389/fmed.2025.1537139 (PMC12098638; doi:10.3389/fmed.2025.1537139)
Supplement: Supplementary file 1 [file Table_1.docx]

**Supplementary Material**

**Unveiling the molecular mechanisms of Stigmasterol on Diabetic Retinopathy: BNM Framework Construction and Experimental Validation**

Hongrong Zhang^#^, Yufan Li^#^, Qi Xu^#,*^ and Zhaohui Fang^*^

Anhui University of Chinese Medicine, Hefei. 230012. China.

* Corresponding authors: xuqiahtcm@yeah.net and fangzhaohui1111@163.com

# These authors contributed equally to this paper and should be considered co-first authors.

**Supplementary Tables**

| **physicochemical descriptors** | **feature importance** | **physicochemical descriptors** | **feature importance** |
| --- | --- | --- | --- |
| SMR_VSA4 | 0.035422161 | VSA_EState1 | 0.005962659 |
| Chi4n | 0.035306184 | PEOE_VSA1 | 0.005958246 |
| SlogP_VSA4 | 0.031124687 | SlogP_VSA11 | 0.00592549 |
| NumHeteroatoms | 0.030497661 | SlogP_VSA8 | 0.005871947 |
| Kappa3 | 0.026810994 | Chi0n | 0.005774438 |
| SlogP_VSA5 | 0.026415604 | RingCount | 0.005769374 |
| Chi3n | 0.025093025 | Chi0v | 0.005623916 |
| Chi4v | 0.024683486 | VSA_EState5 | 0.005425054 |
| VSA_EState7 | 0.024284822 | EState_VSA9 | 0.005388954 |
| NumSaturatedCarbocycles | 0.022187311 | BalabanJ | 0.005223452 |
| NumValenceElectrons | 0.022007005 | MaxPartialCharge | 0.005150505 |
| Chi3v | 0.019318303 | NumAromaticHeterocycles | 0.005097457 |
| NumAliphaticCarbocycles | 0.01773969 | SMR_VSA1 | 0.005053797 |
| SMR_VSA10 | 0.017462976 | SMR_VSA5 | 0.005013651 |
| PEOE_VSA8 | 0.016956866 | PEOE_VSA11 | 0.004618792 |
| Kappa1 | 0.016792097 | SMR_VSA7 | 0.004581265 |
| VSA_EState8 | 0.016287181 | NHOHCount | 0.004265785 |
| Kappa2 | 0.015955278 | BertzCT | 0.004233377 |
| Chi0 | 0.01449521 | MinEStateIndex | 0.004184269 |
| SlogP_VSA3 | 0.013686693 | SlogP_VSA6 | 0.004160356 |
| EState_VSA10 | 0.013586297 | fr_benzene | 0.004059852 |
| NumAliphaticRings | 0.013543403 | MinAbsPartialCharge | 0.003846002 |
| fr_phenol_noOrthoHbond | 0.013293307 | fr_allylic_oxid | 0.003796981 |
| LabuteASA | 0.013248559 | fr_ester | 0.003796342 |
| FpDensityMorgan3 | 0.01299117 | PEOE_VSA9 | 0.003783455 |
| PEOE_VSA10 | 0.012825306 | EState_VSA2 | 0.003691377 |
| HeavyAtomMolWt | 0.012546277 | EState_VSA7 | 0.003307661 |
| Chi2n | 0.012337888 | PEOE_VSA6 | 0.003169724 |
| Chi1v | 0.011986248 | FpDensityMorgan1 | 0.002926417 |
| NumSaturatedRings | 0.011339596 | Chi2v | 0.002907648 |
| SMR_VSA9 | 0.011113072 | HallKierAlpha | 0.002881075 |
| PEOE_VSA7 | 0.010713231 | MaxAbsPartialCharge | 0.002822226 |
| Chi1n | 0.010053476 | fr_Al_OH | 0.002367513 |
| fr_bicyclic | 0.00988194 | MinAbsEStateIndex | 0.00228816 |
| EState_VSA5 | 0.009200956 | NumAromaticCarbocycles | 0.002278834 |
| MaxEStateIndex | 0.00920006 | fr_ketone | 0.002080374 |
| VSA_EState3 | 0.009102252 | fr_piperdine | 0.001969584 |
| Chi1 | 0.009047453 | NumHAcceptors | 0.001960171 |
| EState_VSA3 | 0.009036344 | fr_Al_OH_noTert | 0.001926341 |
| MaxAbsEStateIndex | 0.008875399 | fr_Nhpyrrole | 0.001832228 |
| HeavyAtomCount | 0.008845261 | fr_para_hydroxylation | 0.001823014 |
| ExactMolWt | 0.008708159 | EState_VSA6 | 0.001806698 |
| fr_Ar_OH | 0.008543605 | fr_methoxy | 0.001650032 |
| NumRotatableBonds | 0.008538086 | NumHDonors | 0.001385339 |
| fr_phenol | 0.008417769 | PEOE_VSA14 | 0.00136379 |
| MolLogP | 0.008375176 | MolWt | 0.001071308 |
| FpDensityMorgan2 | 0.008183228 | fr_Ar_N | 0.000863774 |
| EState_VSA1 | 0.008042967 | PEOE_VSA5 | 0.000852369 |
| VSA_EState4 | 0.00746815 | VSA_EState6 | 0.000852369 |
| FractionCSP3 | 0.007232153 | MinPartialCharge | 0.00073735 |
| EState_VSA4 | 0.007154514 | PEOE_VSA13 | 0.000726912 |
| SlogP_VSA1 | 0.006721539 | fr_C_O | 0.000647494 |
| NOCount | 0.006479091 | PEOE_VSA2 | 0.000572606 |
| TPSA | 0.006469671 | fr_aryl_methyl | 0.000467301 |
| SMR_VSA6 | 0.006335165 | fr_ether | 0.00046313 |
| EState_VSA8 | 0.006312197 | NumSaturatedHeterocycles | 0.000397436 |
| VSA_EState2 | 0.006310487 | NumAromaticRings | 0.000326431 |
| qed | 0.006176555 | NumAliphaticHeterocycles | 0.000163623 |
| SlogP_VSA2 | 0.005992925 | VSA_EState9 | 0.000103936 |
| MolMR | 0.005988841 | SMR_VSA3 | 5.33E-06 |

**Supplementary Table 1**.
