# Supplementary figures and images for: Unveiling the molecular mechanisms of stigmasterol on diabetic retinopathy: BNM framework construction and experimental validation
Source: Front Med (Lausanne). 2025 May 9;12:1537139. doi: 10.3389/fmed.2025.1537139 (PMC12098638; doi:10.3389/fmed.2025.1537139)

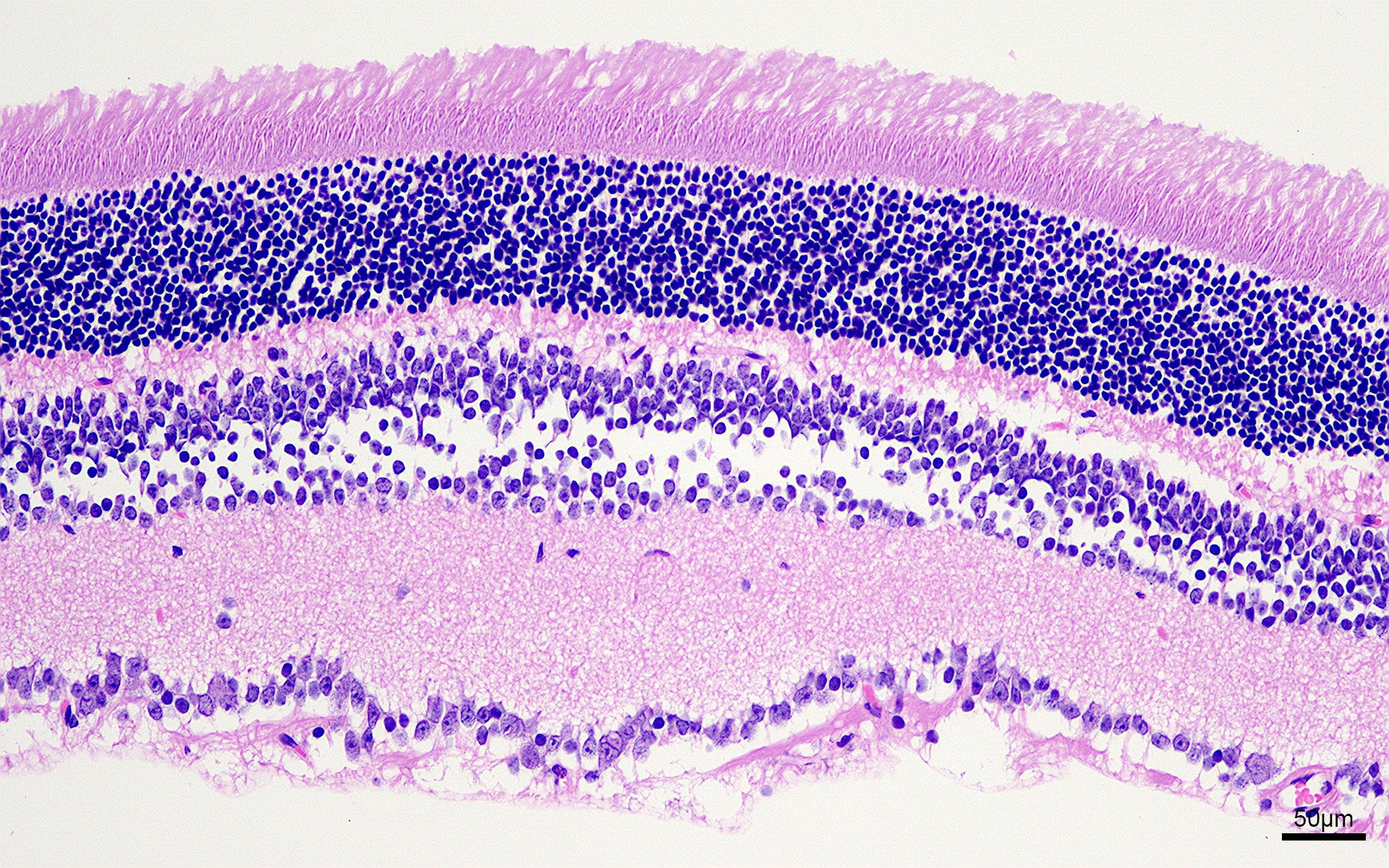

Supplement: Supplementary file 2 [file Data_Sheet_1.zip › Supplement_Data/Experimental Data/HE staining of the retina/Chinese medcine 200-1.jpg]

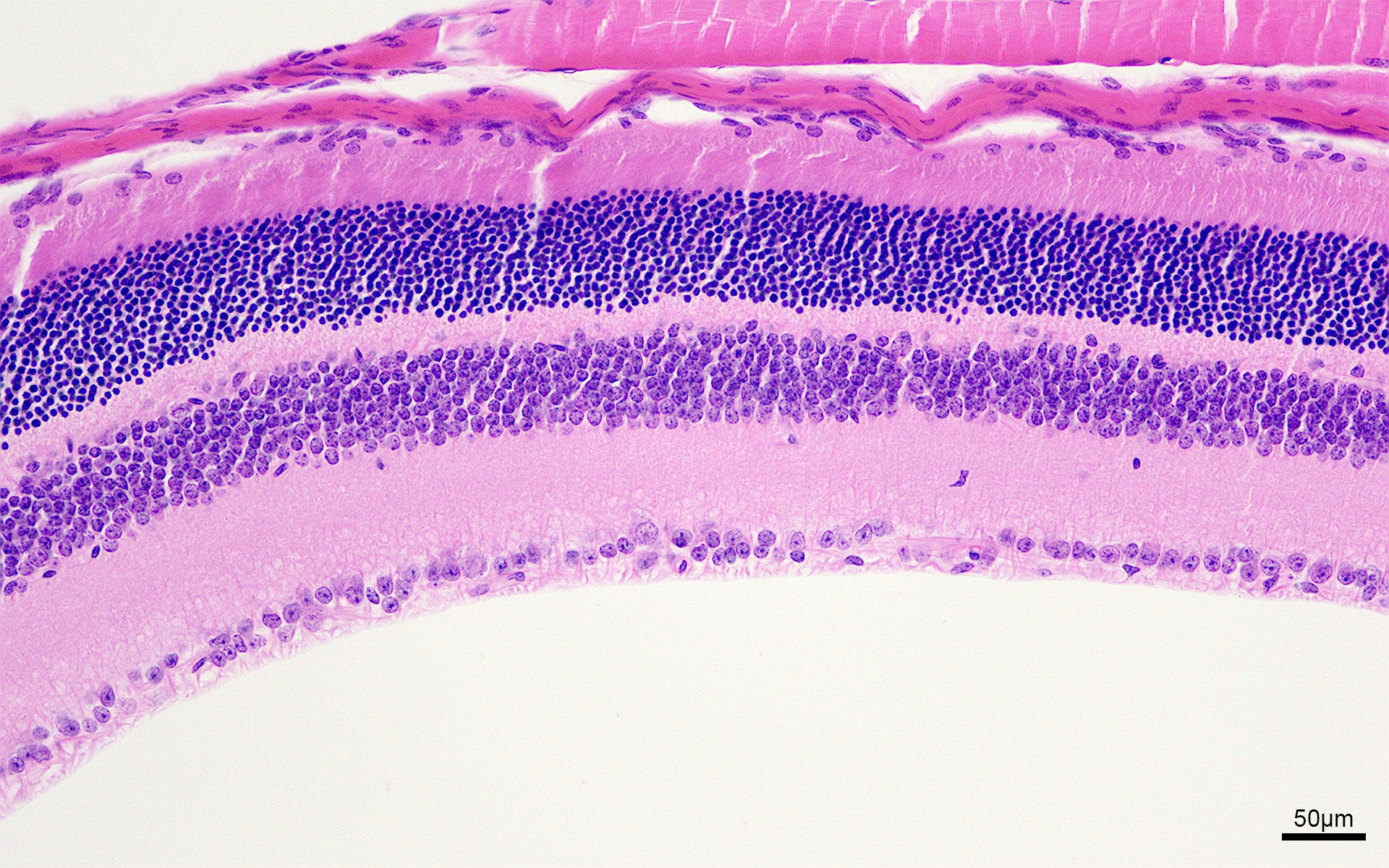

Supplement: Supplementary file 2 [file Data_Sheet_1.zip › Supplement_Data/Experimental Data/HE staining of the retina/control 200-1.jpg]

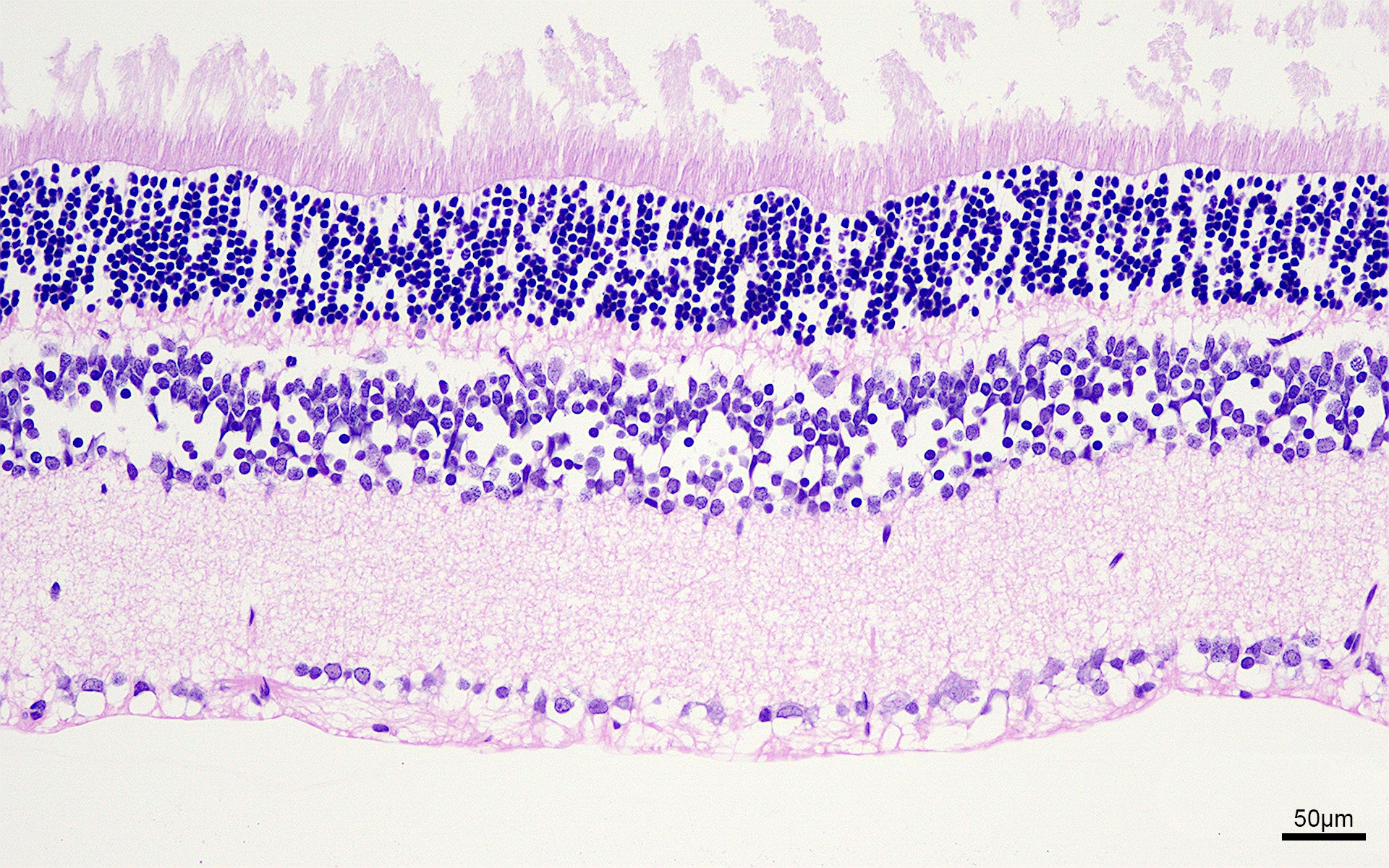

Supplement: Supplementary file 2 [file Data_Sheet_1.zip › Supplement_Data/Experimental Data/HE staining of the retina/model 200-1.jpg]

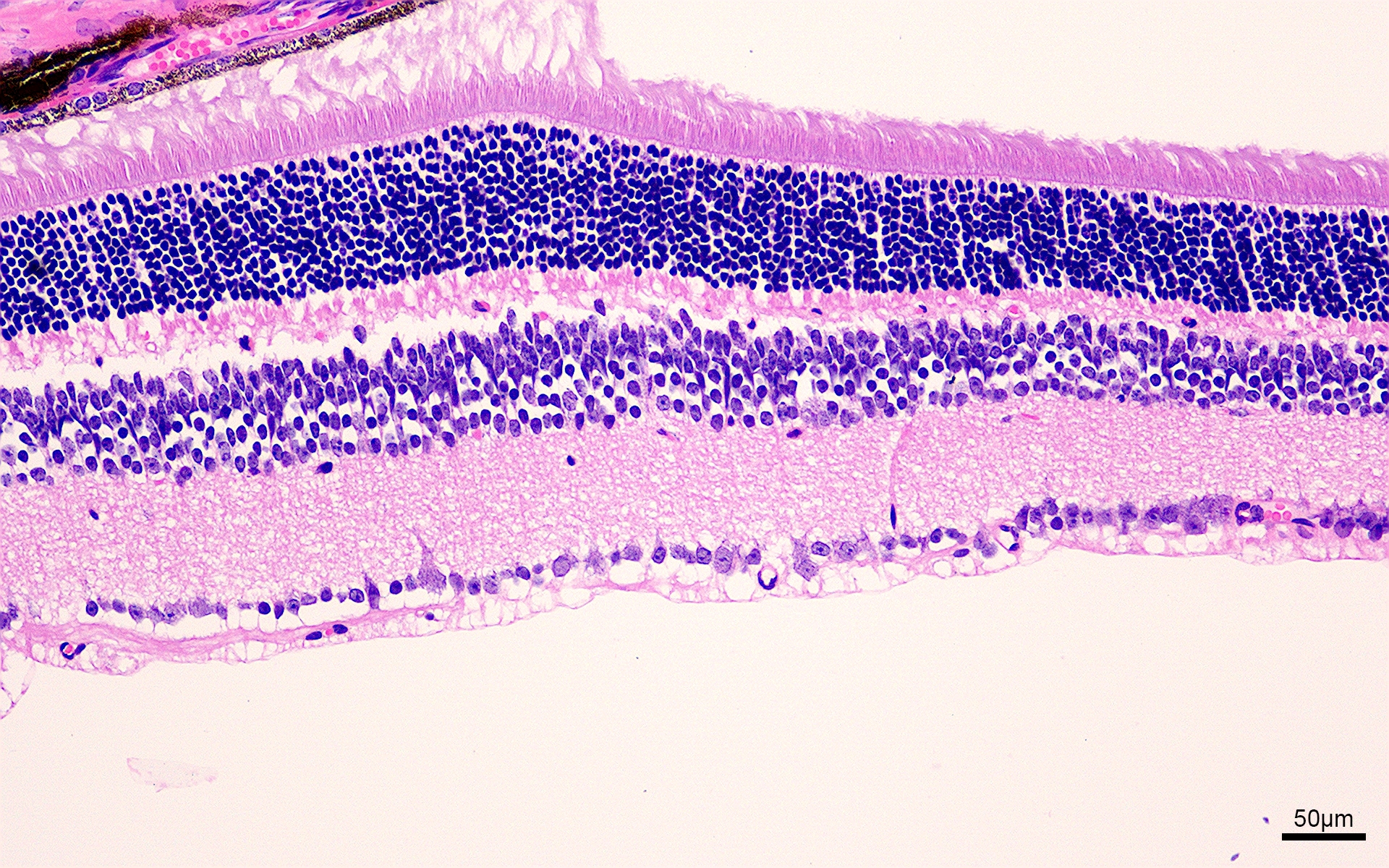

Supplement: Supplementary file 2 [file Data_Sheet_1.zip › Supplement_Data/Experimental Data/HE staining of the retina/positive control 200-1.jpg]

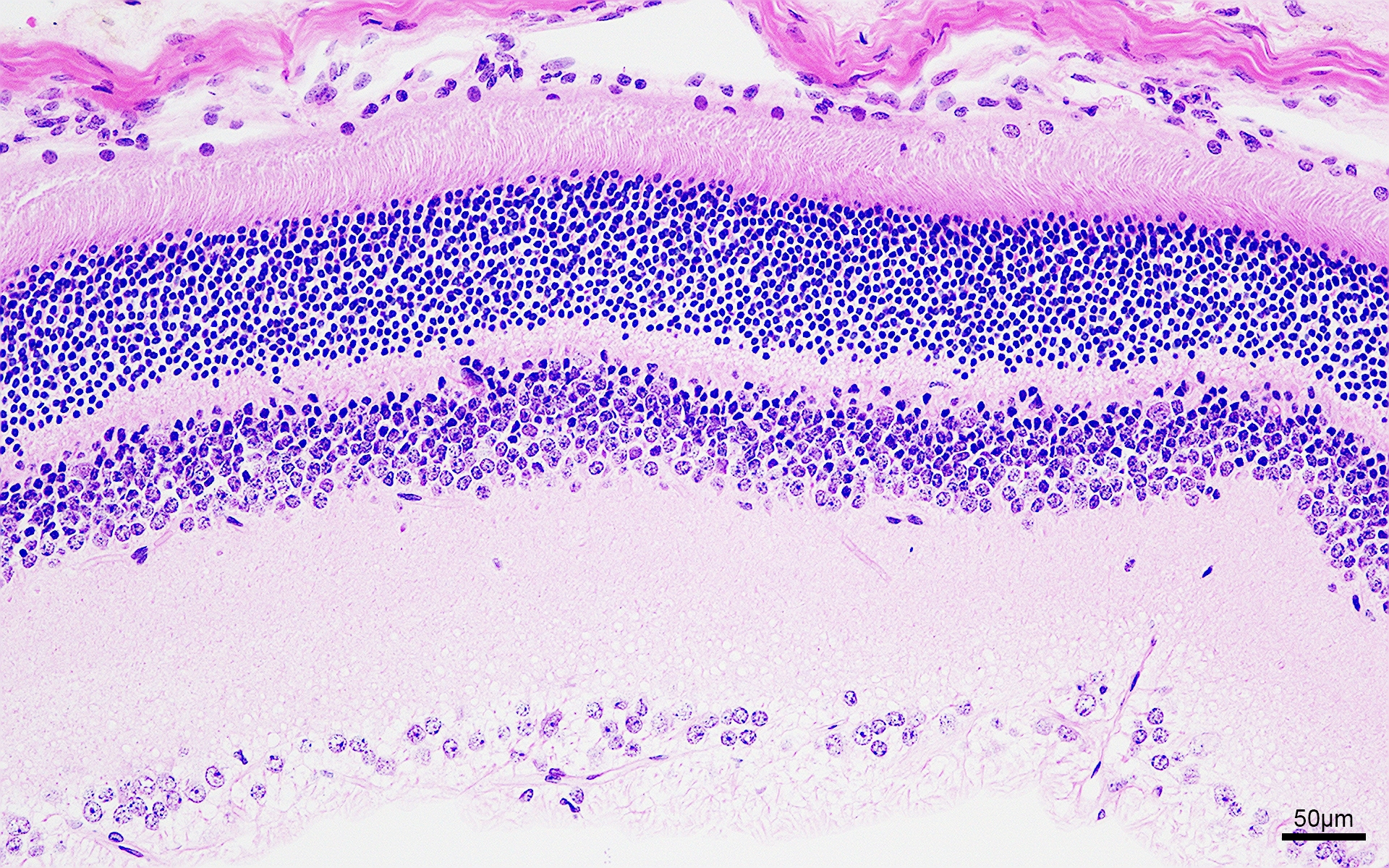

Supplement: Supplementary file 2 [file Data_Sheet_1.zip › Supplement_Data/Experimental Data/HE staining of the retina/stigmasterol 200-1.jpg]

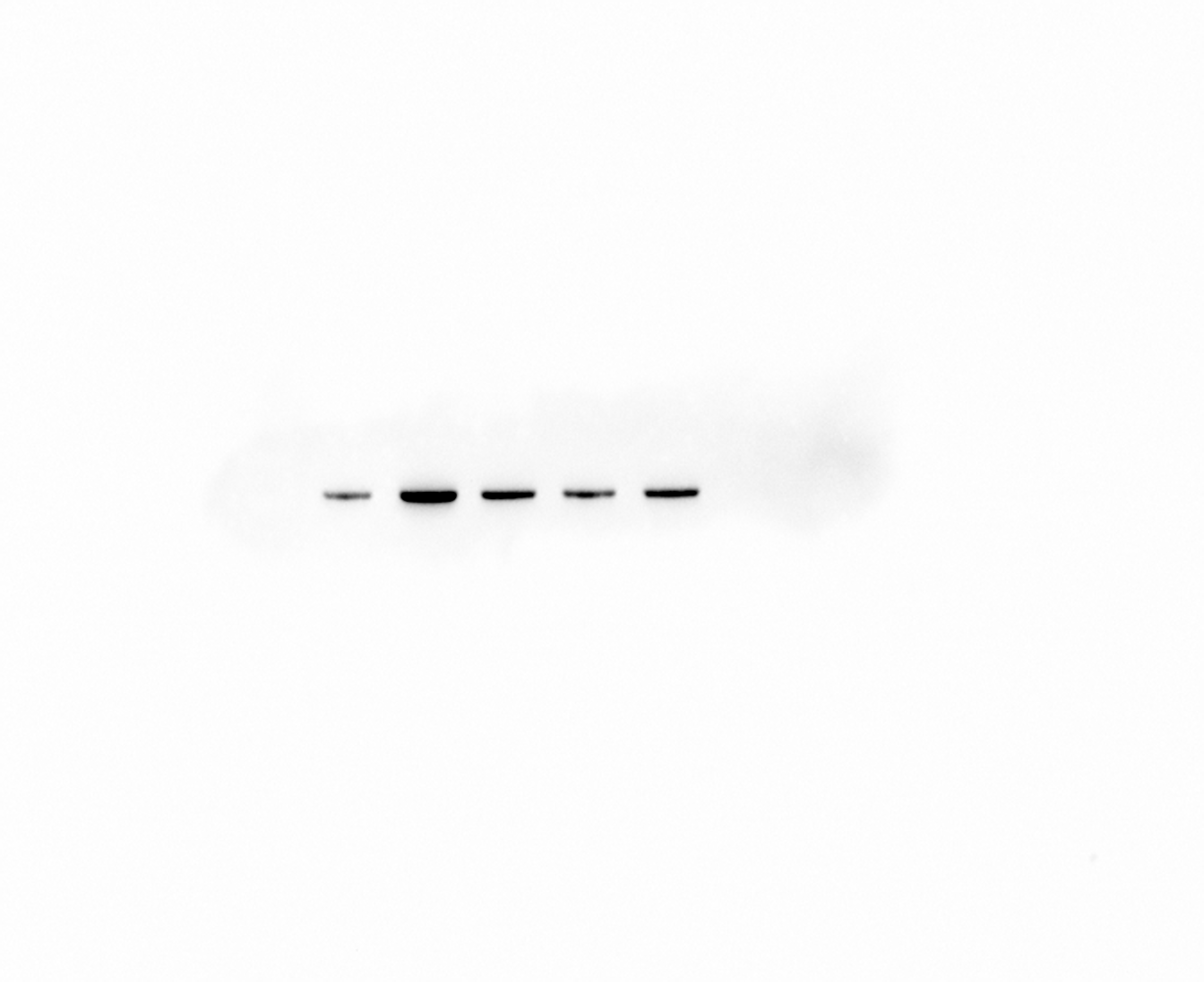

Supplement: Supplementary file 2 [file Data_Sheet_1.zip › Supplement_Data/Experimental Data/WB/liver/Fas-1.Tif]

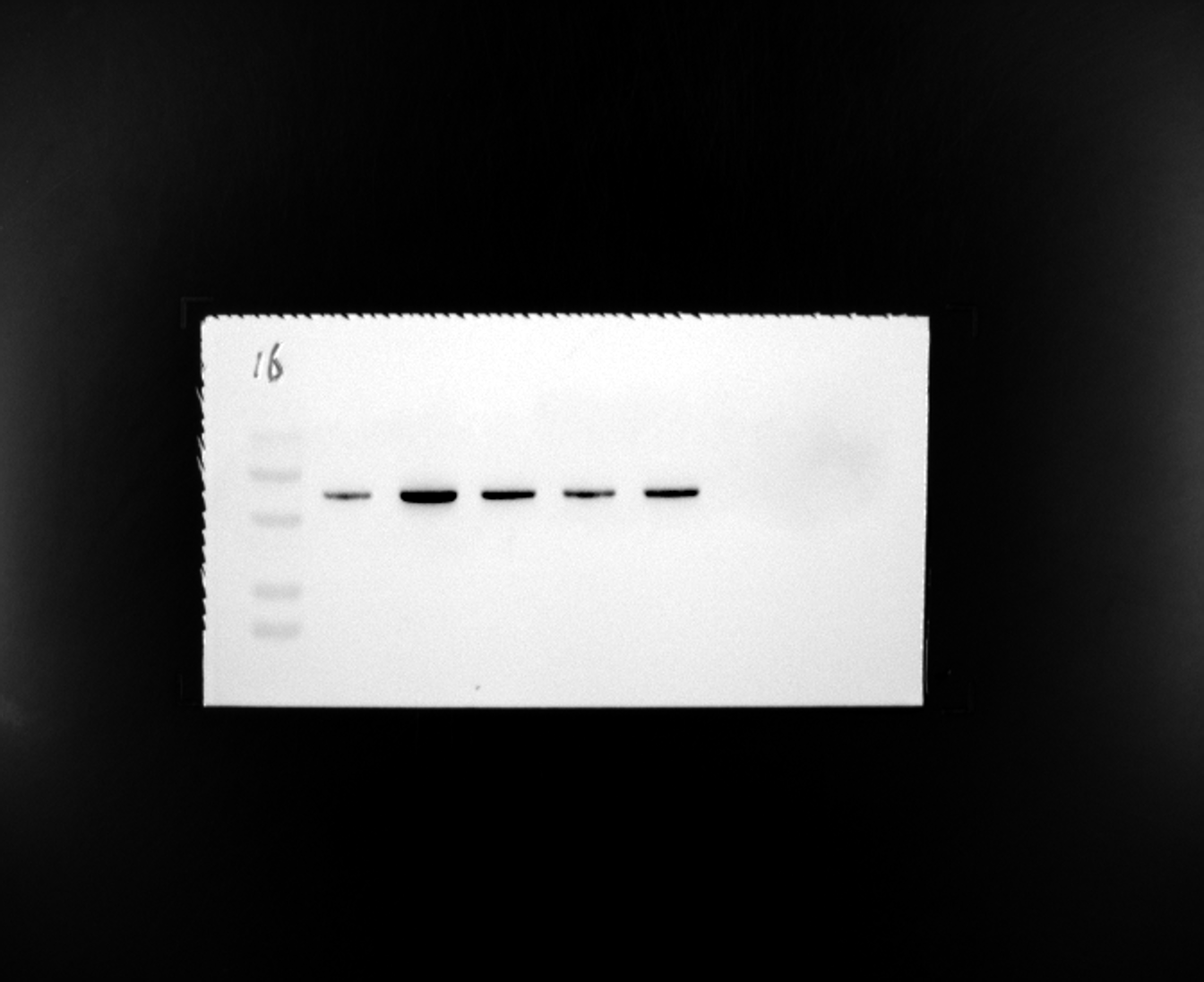

Supplement: Supplementary file 2 [file Data_Sheet_1.zip › Supplement_Data/Experimental Data/WB/liver/Fas-1(M).Tif]

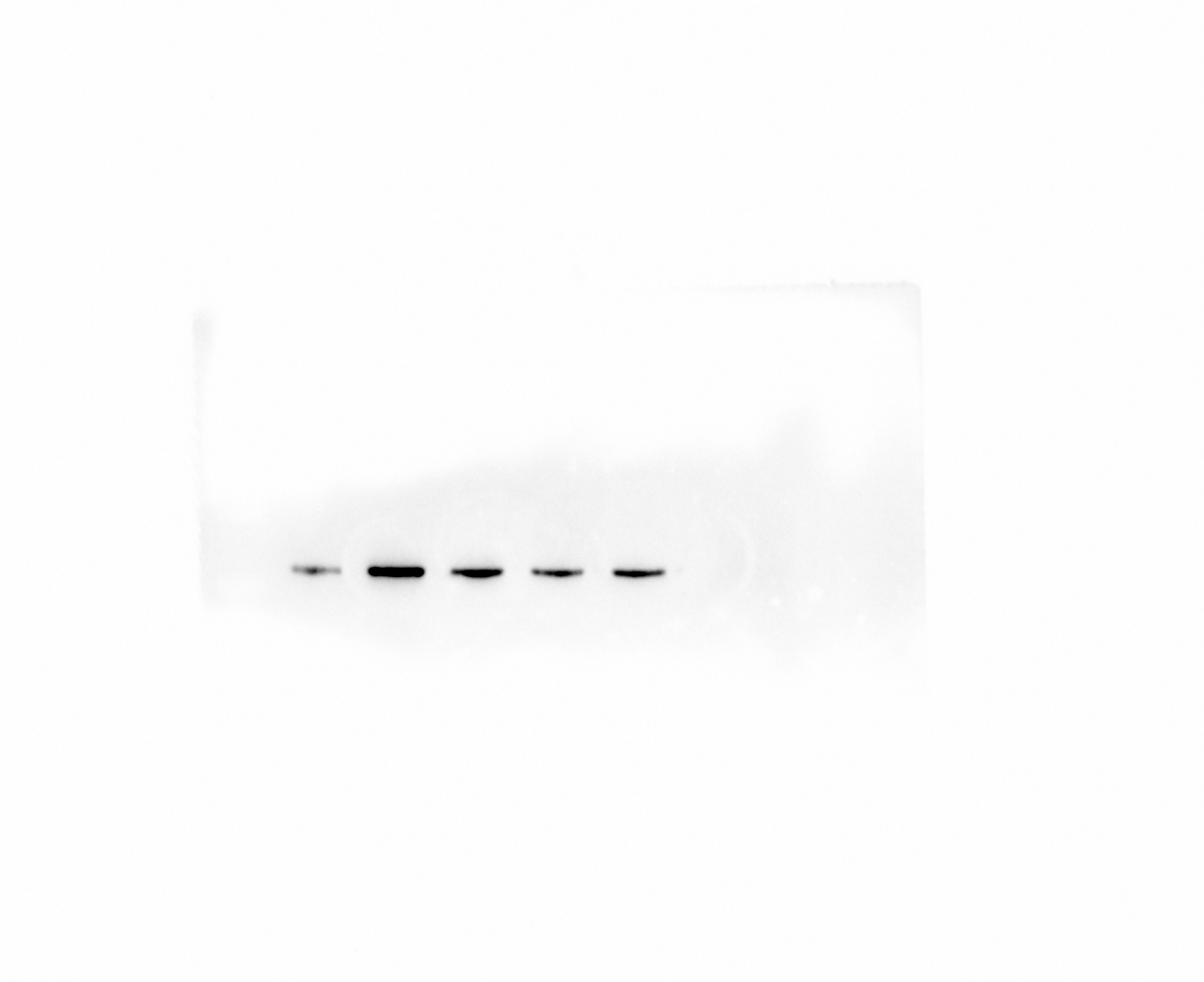

Supplement: Supplementary file 2 [file Data_Sheet_1.zip › Supplement_Data/Experimental Data/WB/liver/G6PASE-1.Tif]

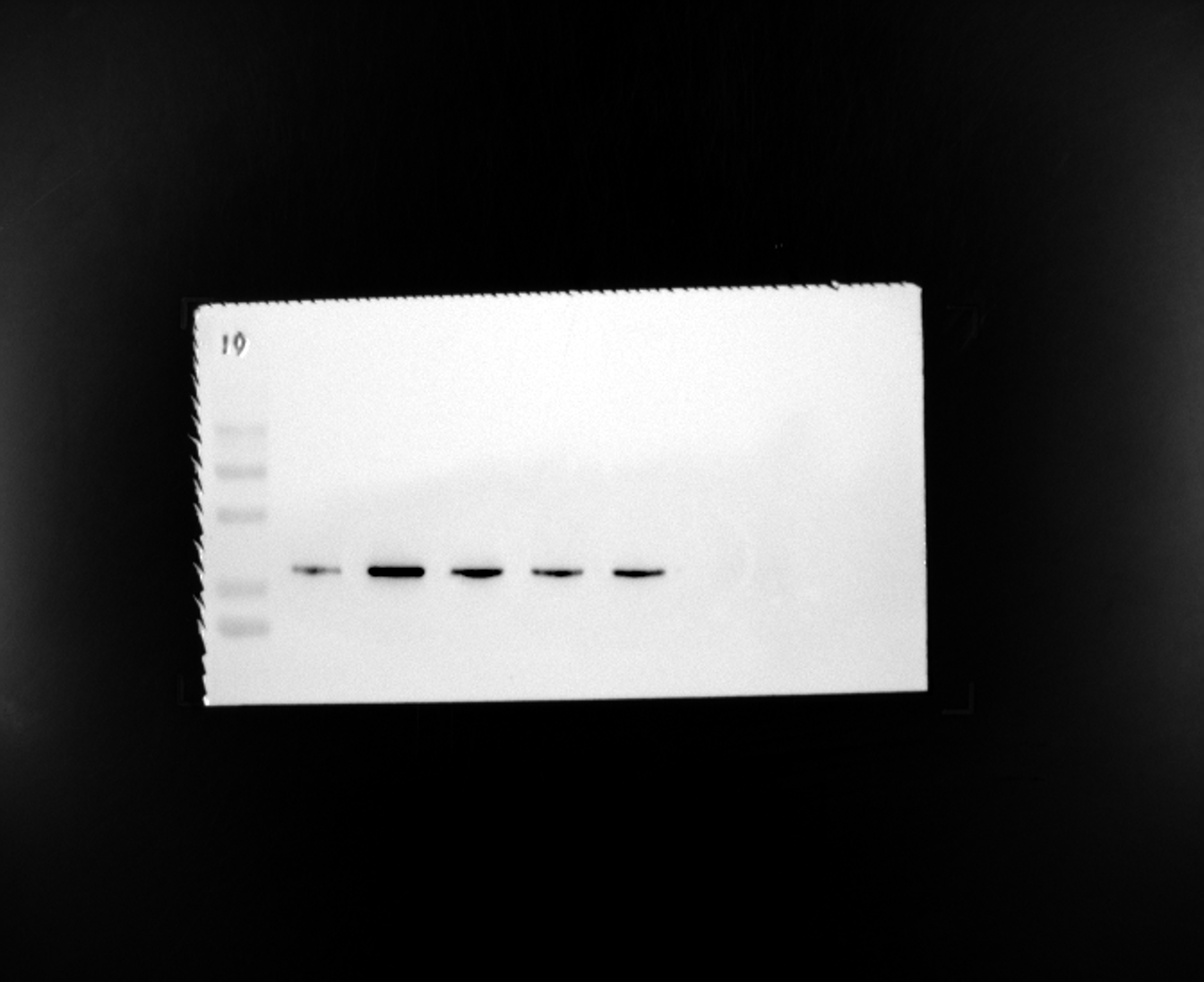

Supplement: Supplementary file 2 [file Data_Sheet_1.zip › Supplement_Data/Experimental Data/WB/liver/G6PASE-1(M).Tif]

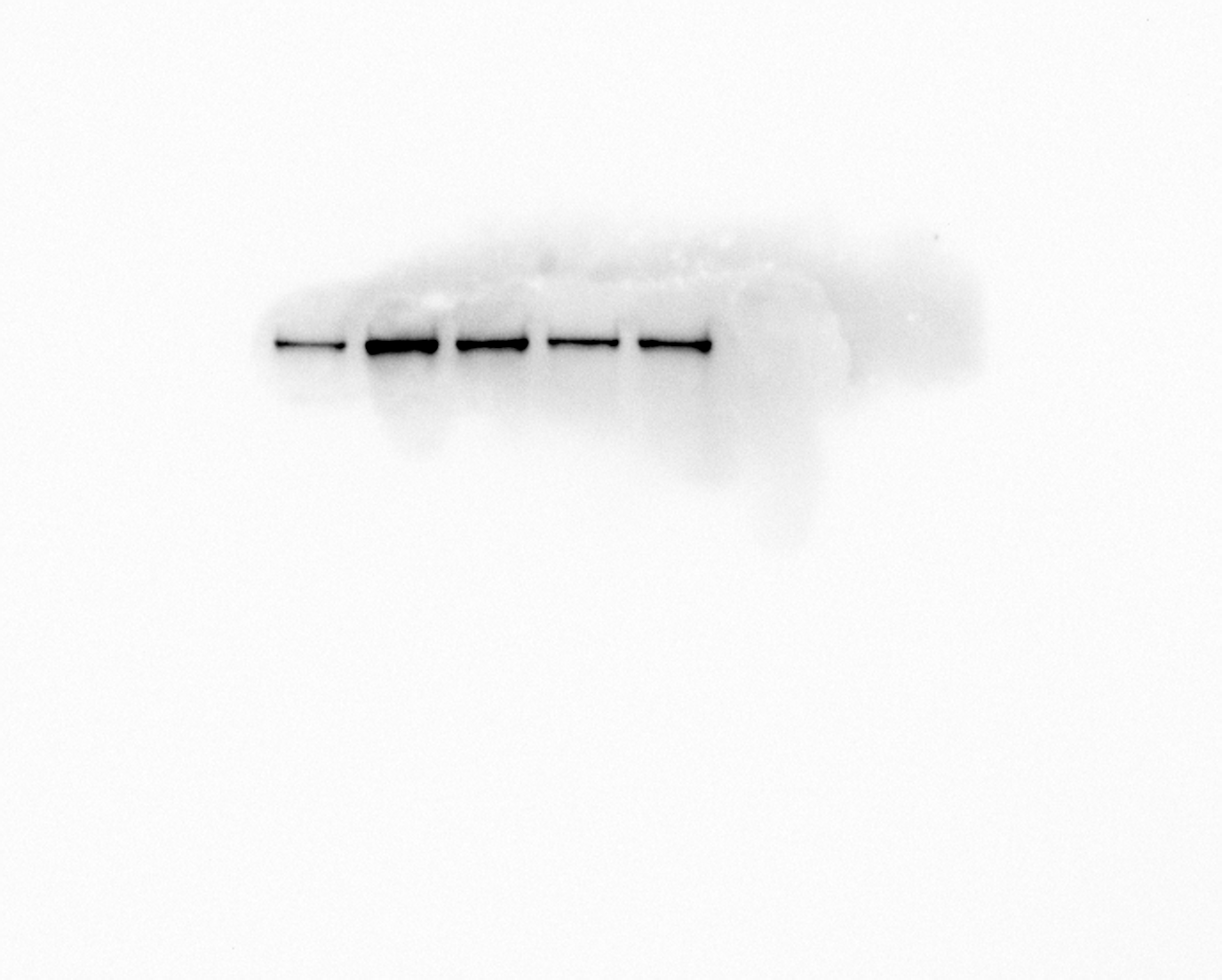

Supplement: Supplementary file 2 [file Data_Sheet_1.zip › Supplement_Data/Experimental Data/WB/liver/PEPCK-1.tif]

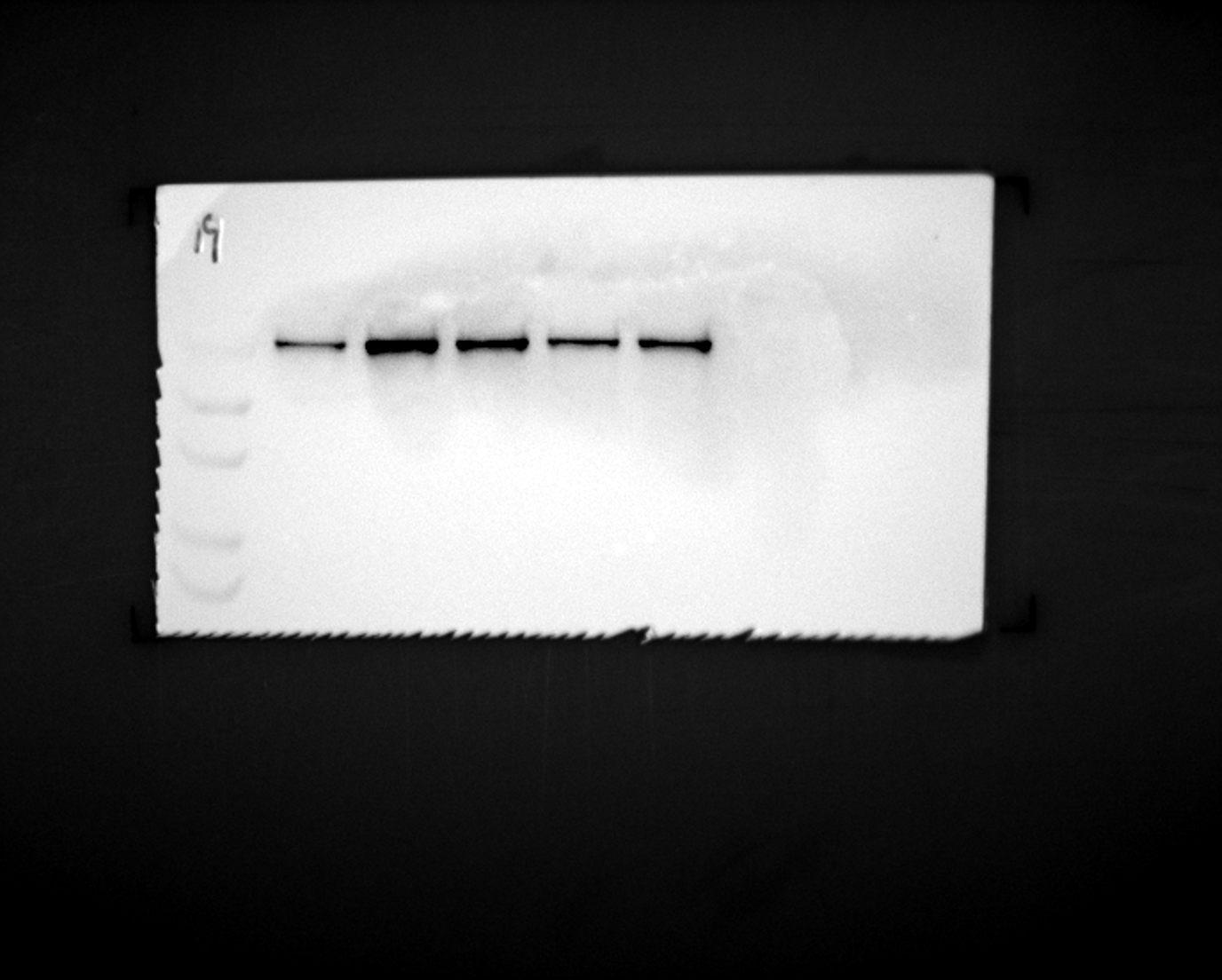

Supplement: Supplementary file 2 [file Data_Sheet_1.zip › Supplement_Data/Experimental Data/WB/liver/PEPCK-1(M).tif]

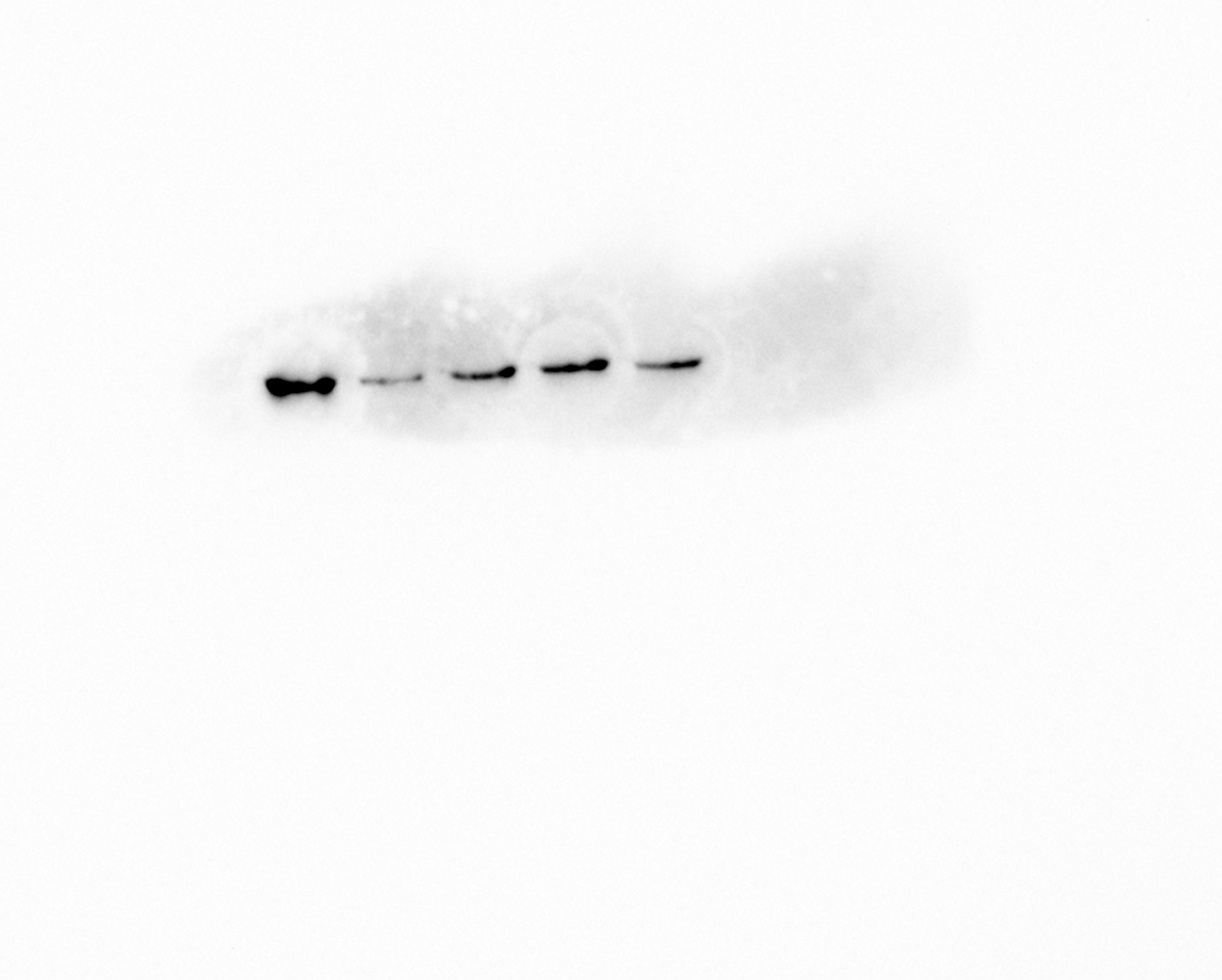

Supplement: Supplementary file 2 [file Data_Sheet_1.zip › Supplement_Data/Experimental Data/WB/liver/PPARG-1.tif]

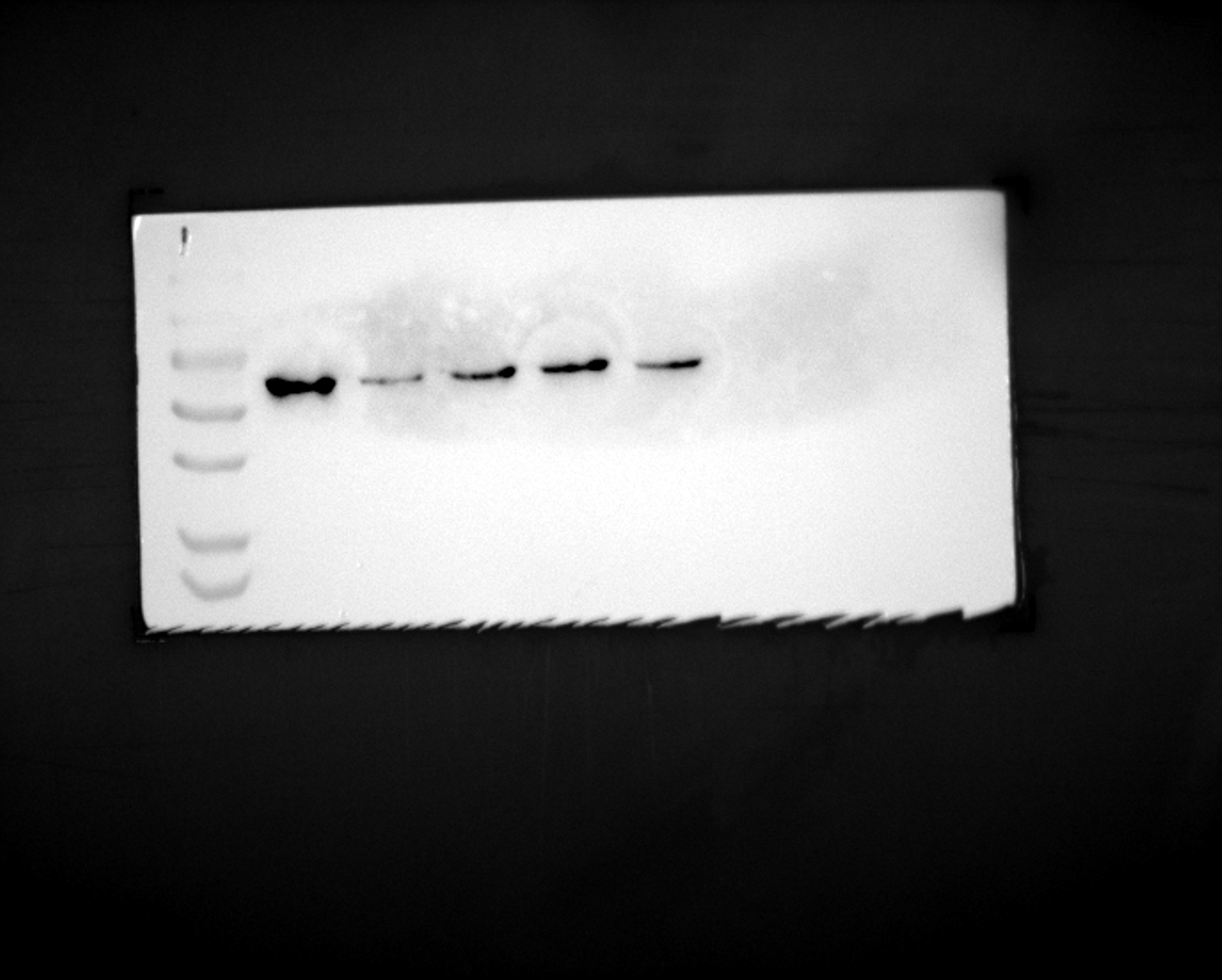

Supplement: Supplementary file 2 [file Data_Sheet_1.zip › Supplement_Data/Experimental Data/WB/liver/PPARG-1(M).tif]

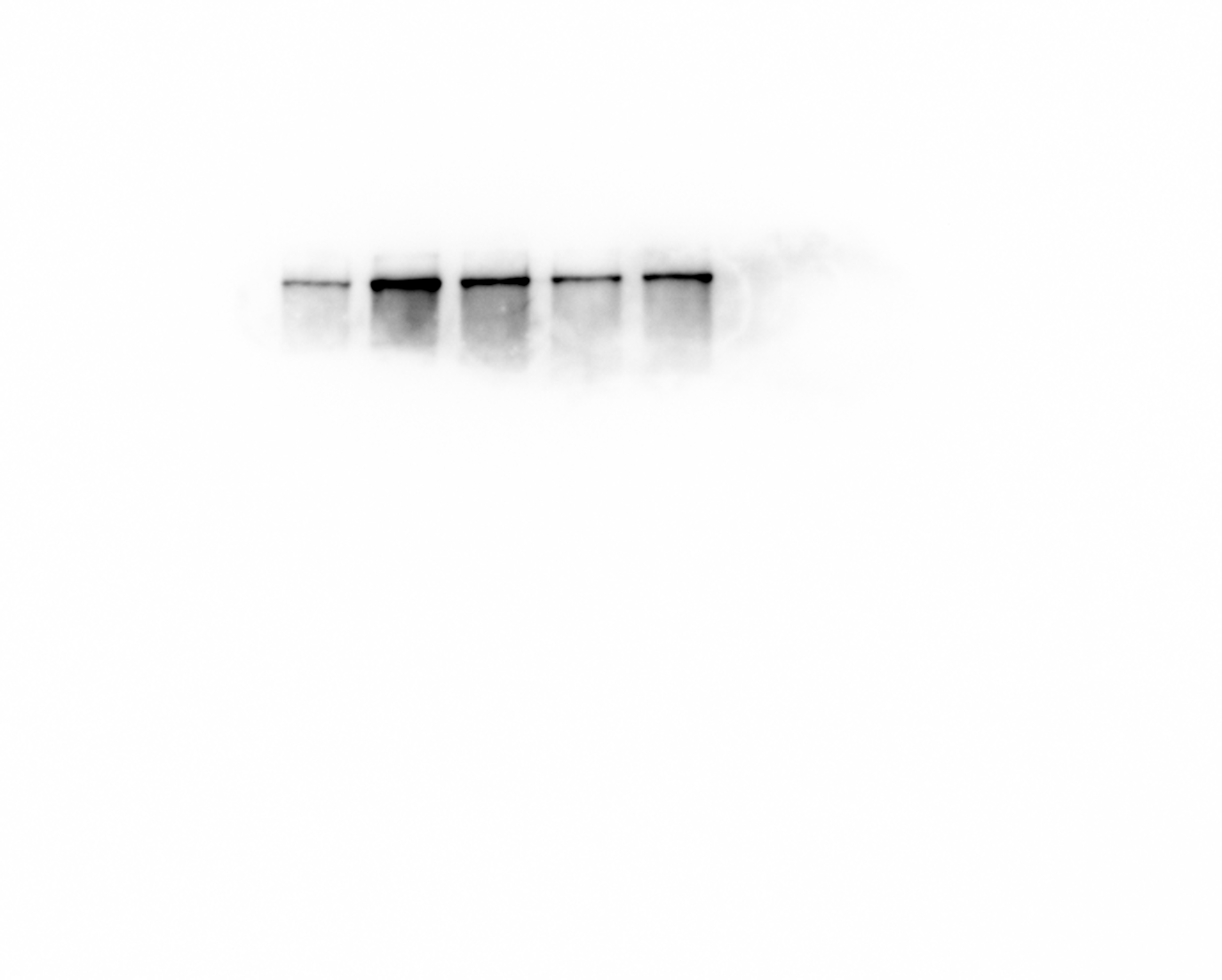

Supplement: Supplementary file 2 [file Data_Sheet_1.zip › Supplement_Data/Experimental Data/WB/liver/SREBP-1C-1.tif]

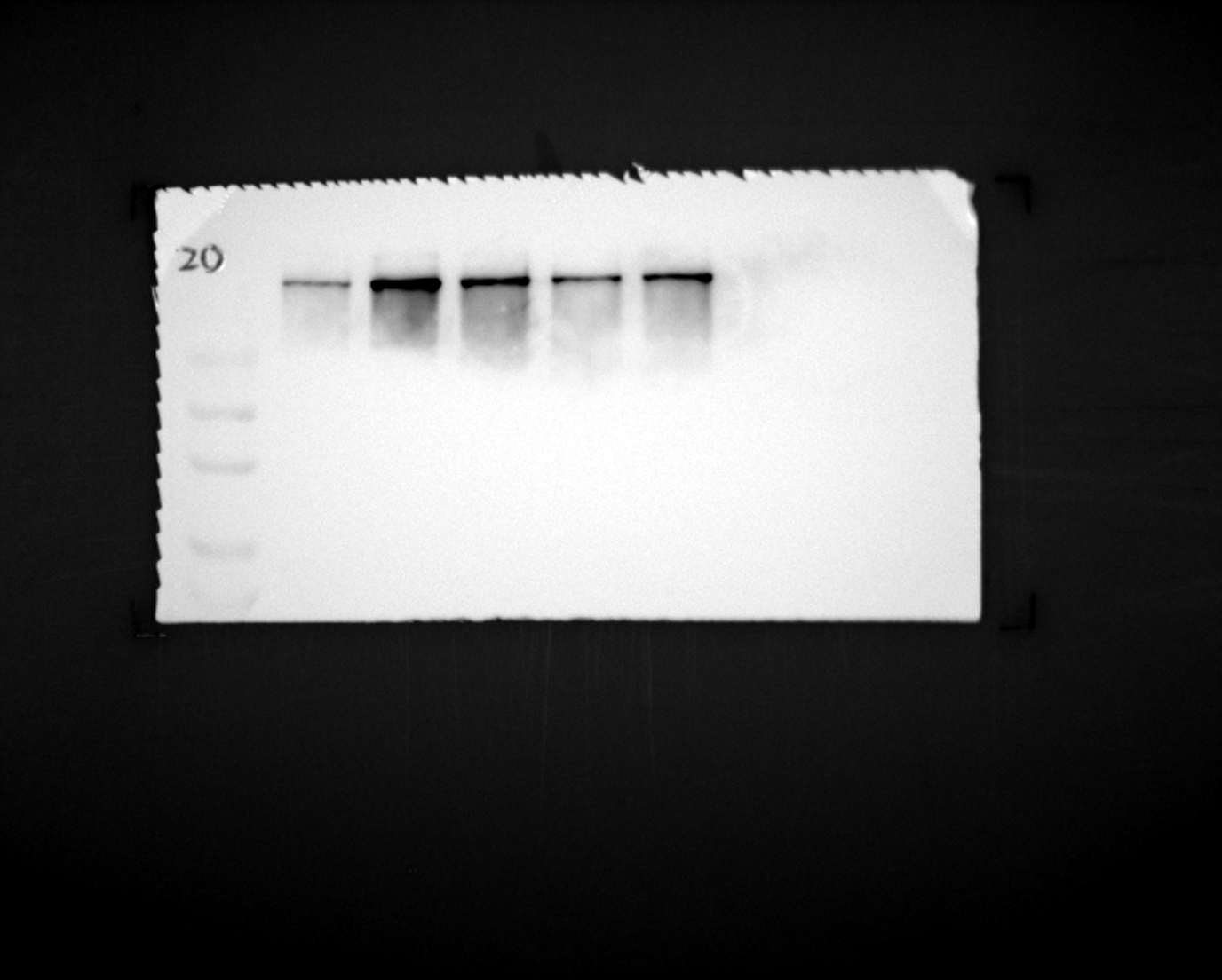

Supplement: Supplementary file 2 [file Data_Sheet_1.zip › Supplement_Data/Experimental Data/WB/liver/SREBP-1C-1(M).tif]

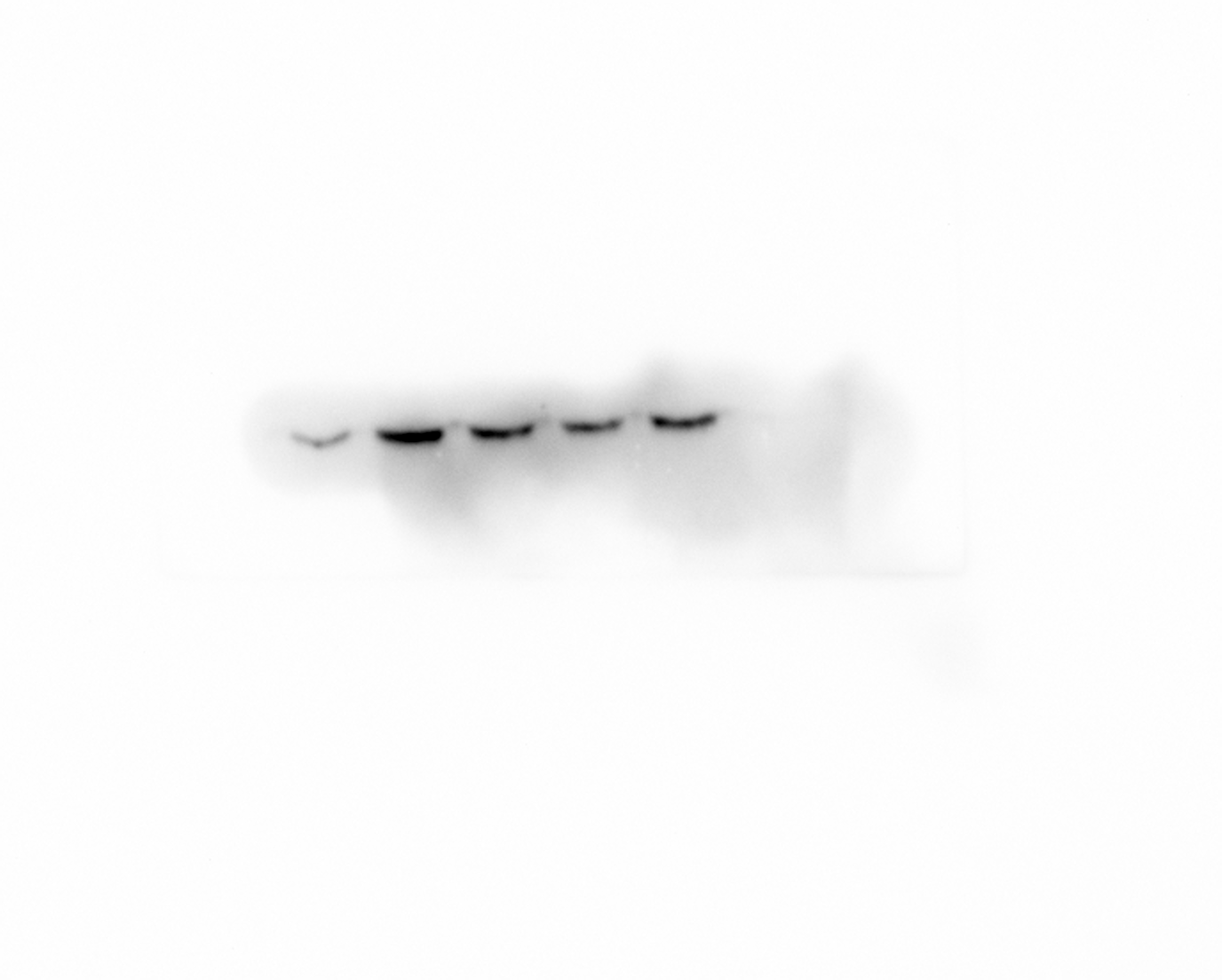

Supplement: Supplementary file 2 [file Data_Sheet_1.zip › Supplement_Data/Experimental Data/WB/liver/VEGFA-1.tif]

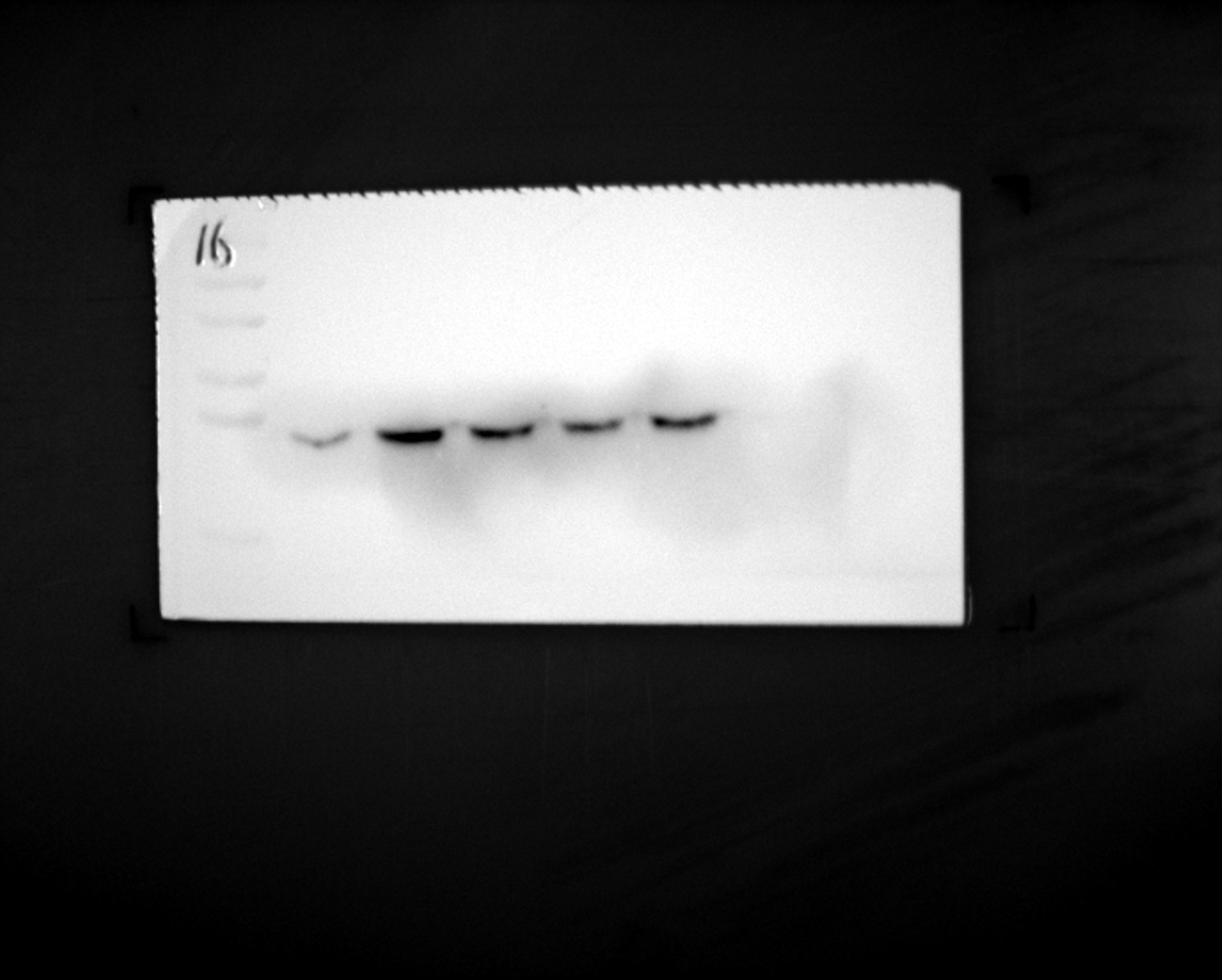

Supplement: Supplementary file 2 [file Data_Sheet_1.zip › Supplement_Data/Experimental Data/WB/liver/VEGFA-1(M).tif]

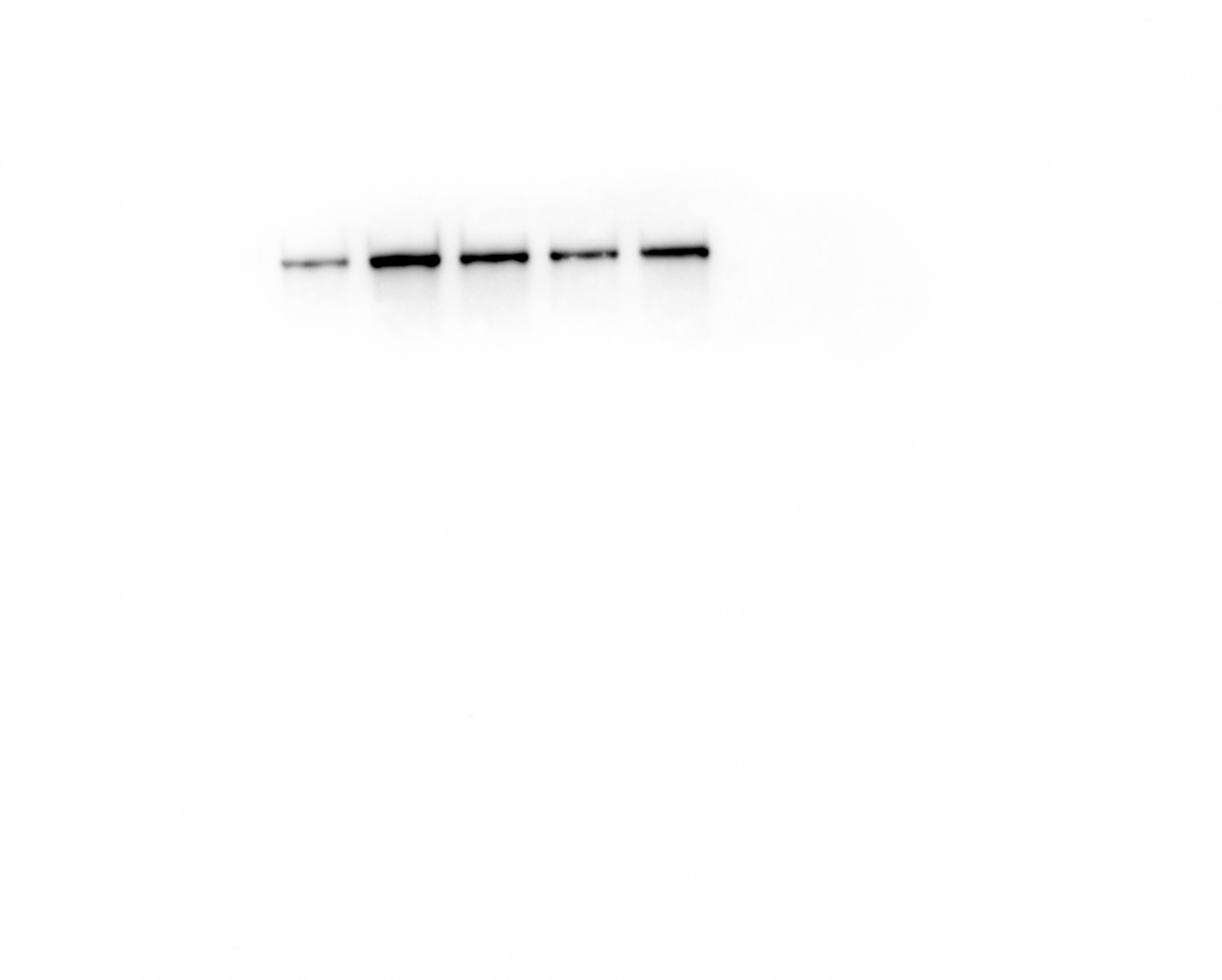

Supplement: Supplementary file 2 [file Data_Sheet_1.zip › Supplement_Data/Experimental Data/WB/liver/VEGFR2-1.tif]

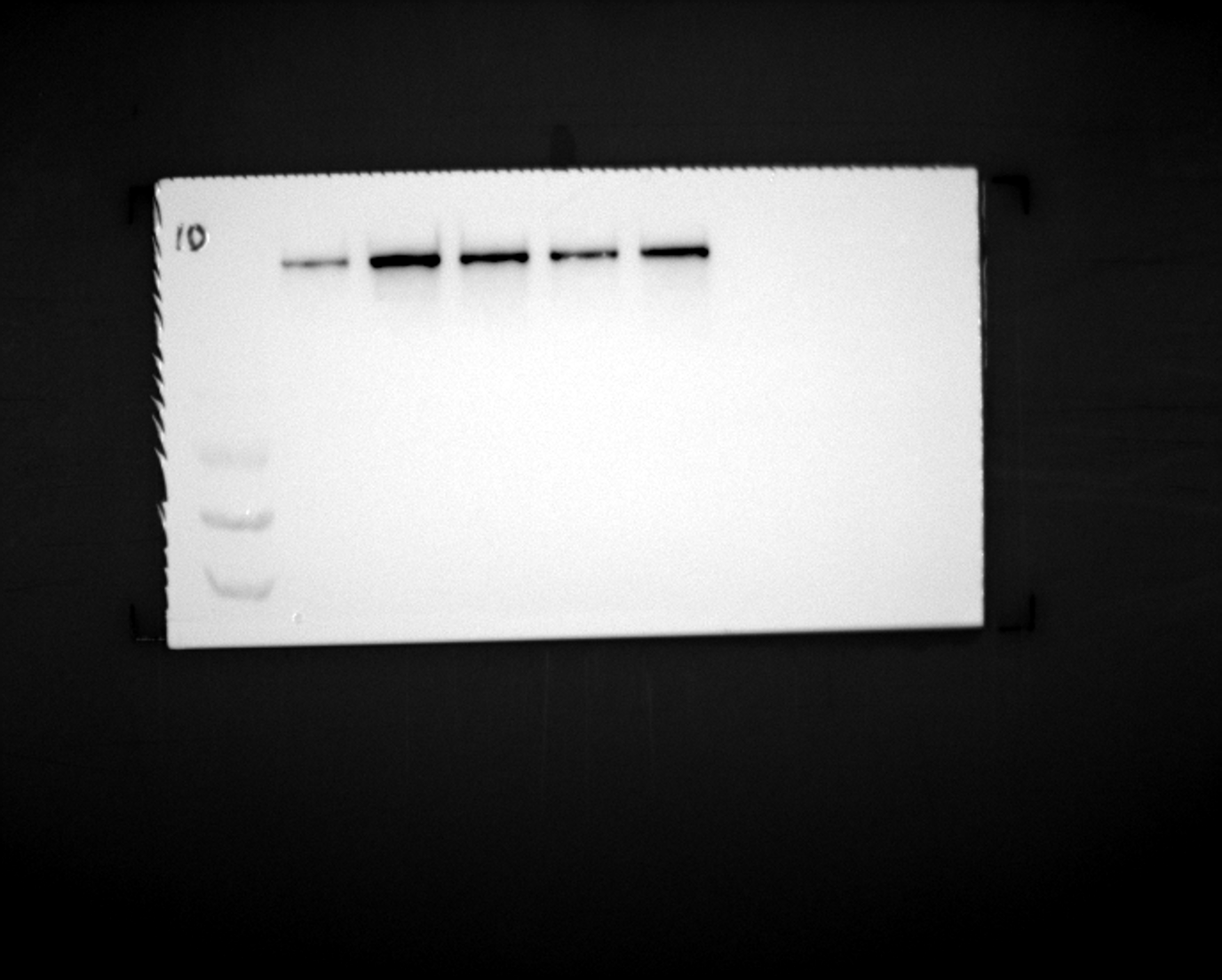

Supplement: Supplementary file 2 [file Data_Sheet_1.zip › Supplement_Data/Experimental Data/WB/liver/VEGFR2-1(M).tif]

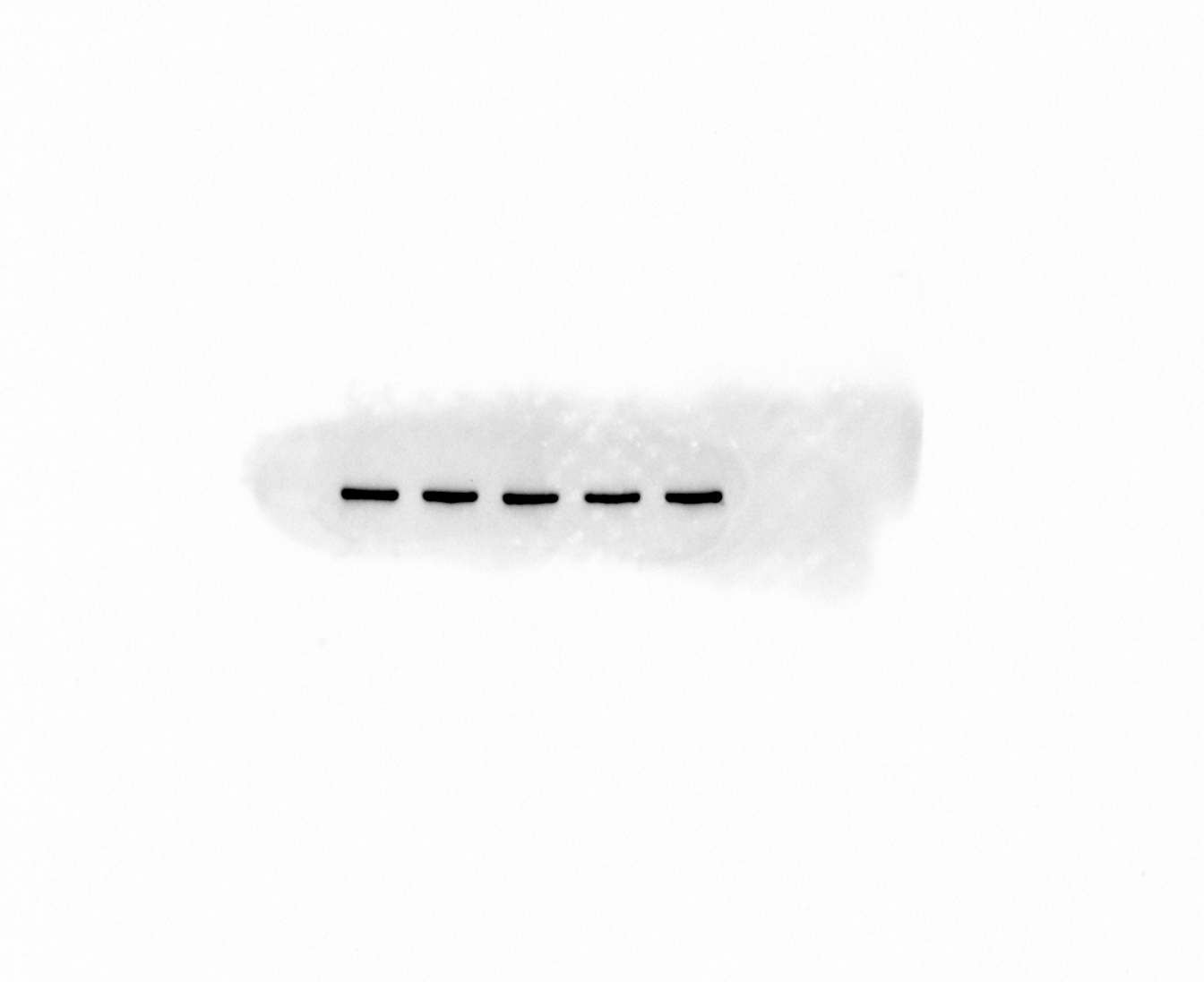

Supplement: Supplementary file 2 [file Data_Sheet_1.zip › Supplement_Data/Experimental Data/WB/liver/β-actin-2.Tif]

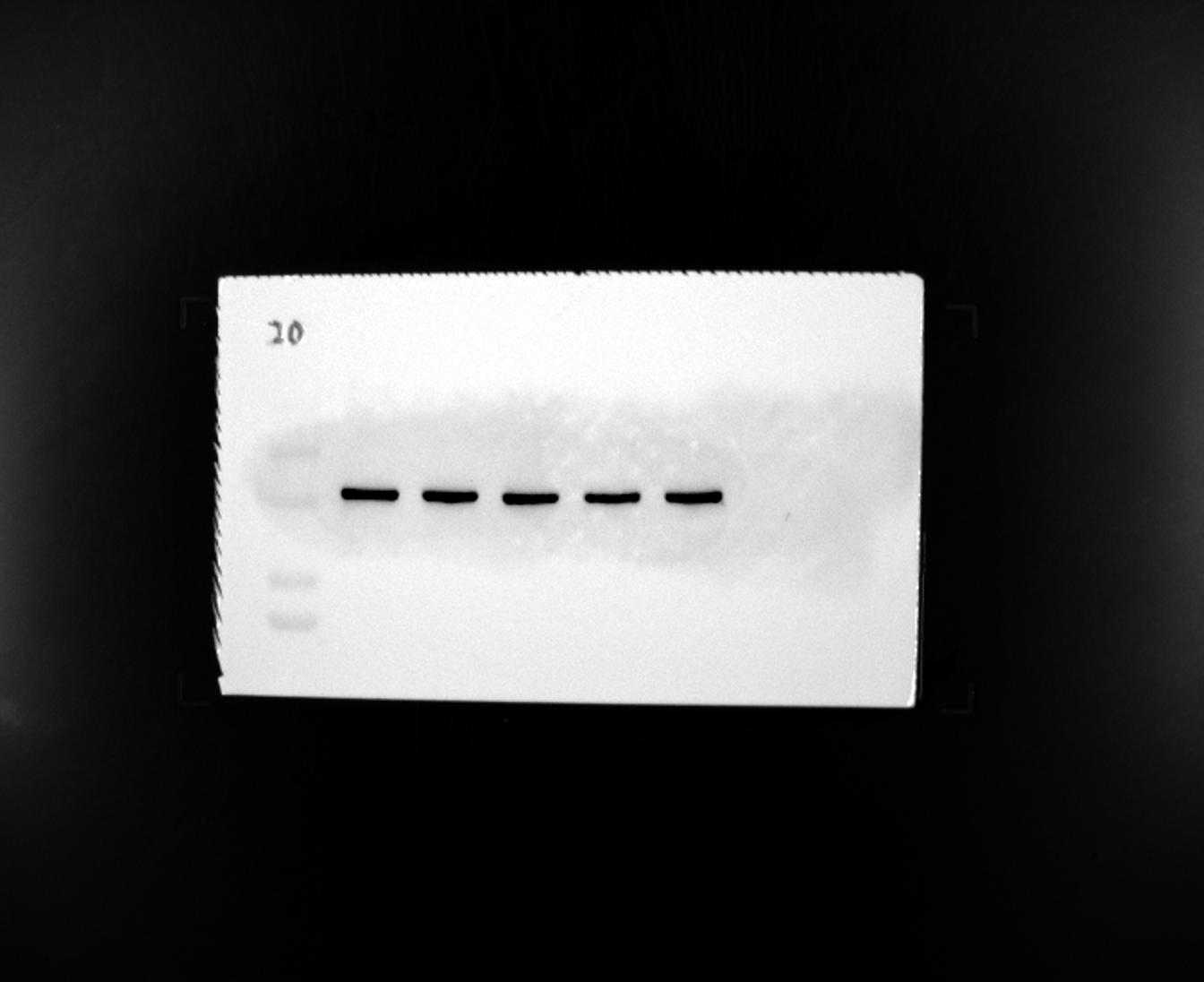

Supplement: Supplementary file 2 [file Data_Sheet_1.zip › Supplement_Data/Experimental Data/WB/liver/β-actin-2(M).Tif]

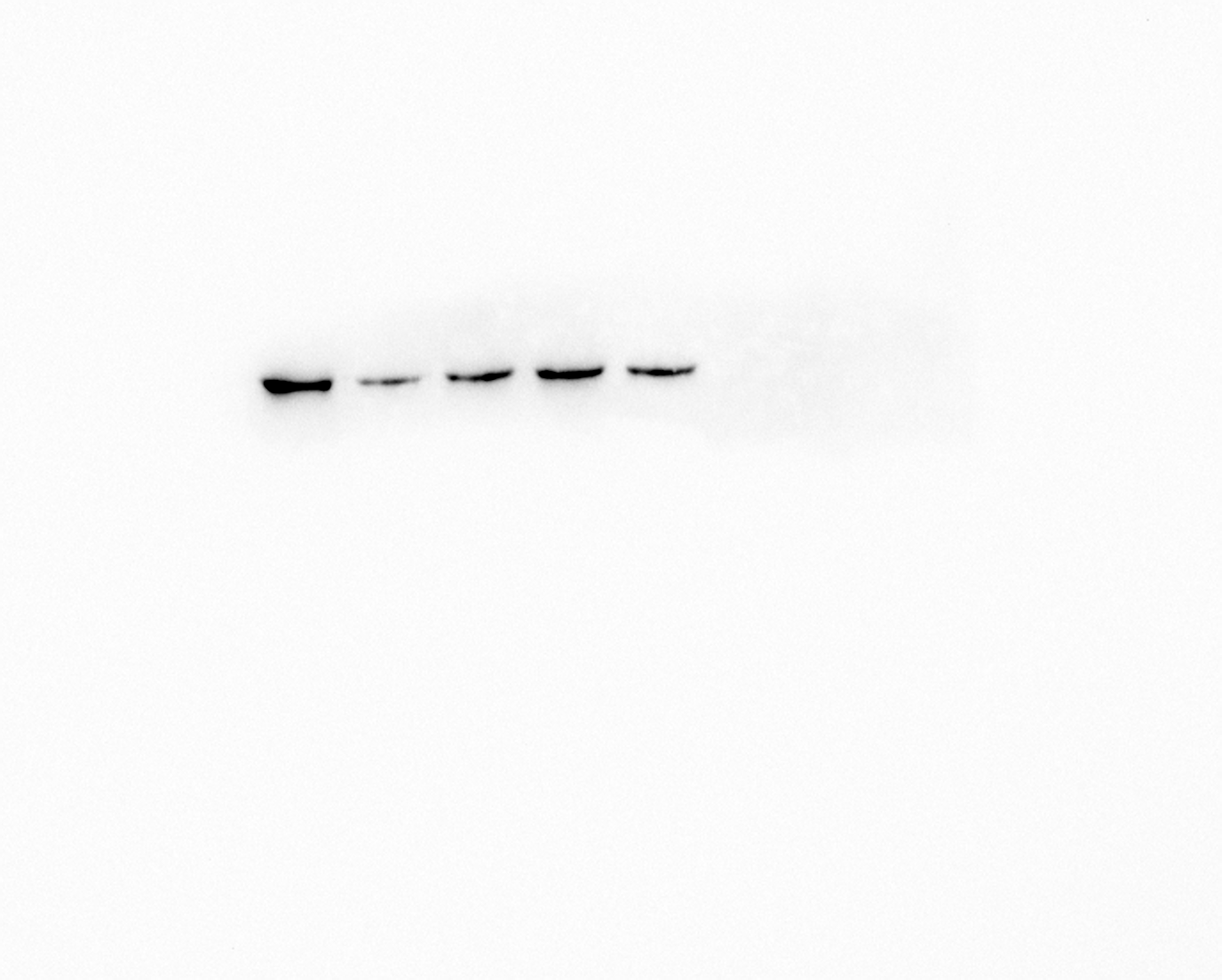

Supplement: Supplementary file 2 [file Data_Sheet_1.zip › Supplement_Data/Experimental Data/WB/retina/PPARG-1.tif]

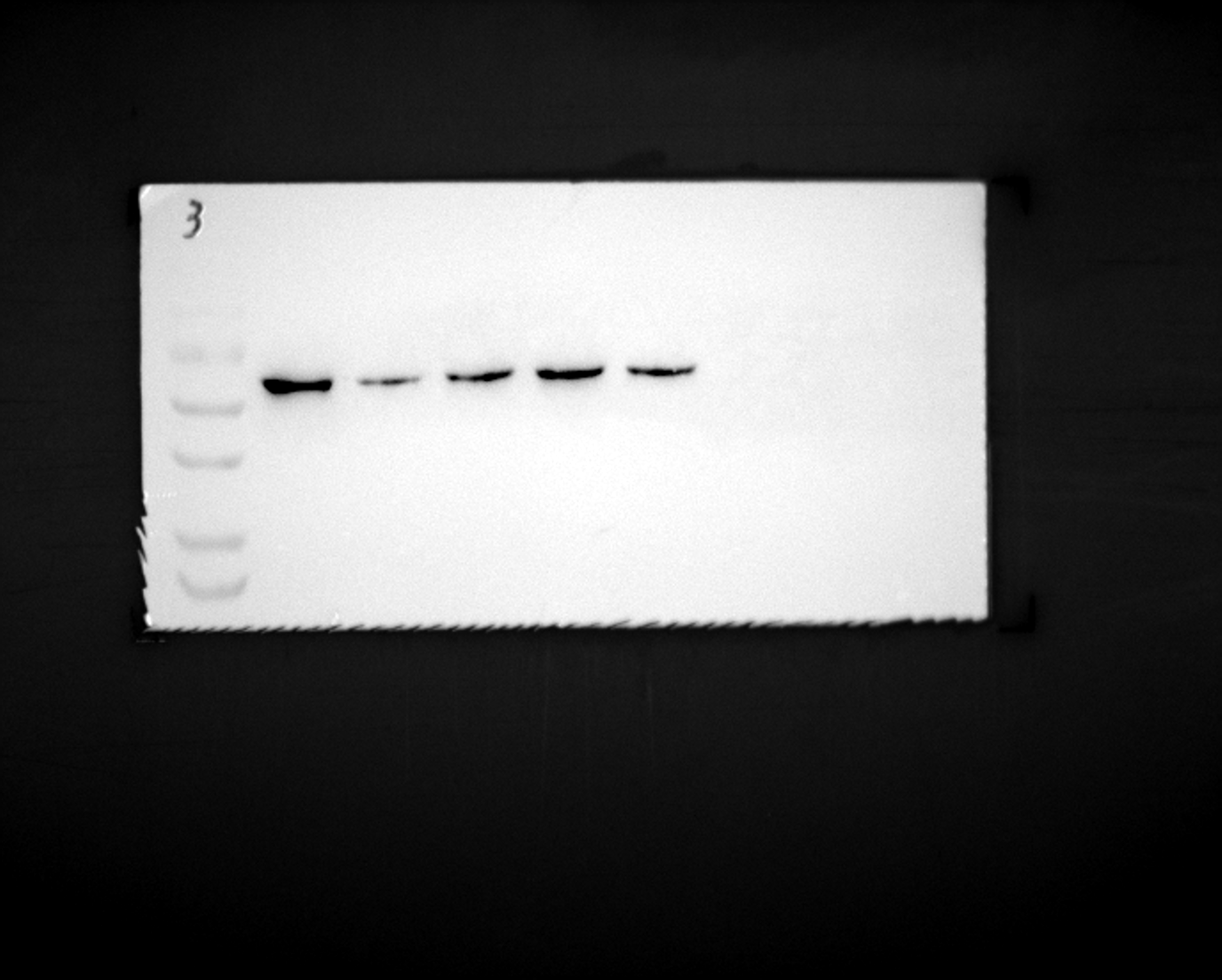

Supplement: Supplementary file 2 [file Data_Sheet_1.zip › Supplement_Data/Experimental Data/WB/retina/PPARG-1(M).tif]

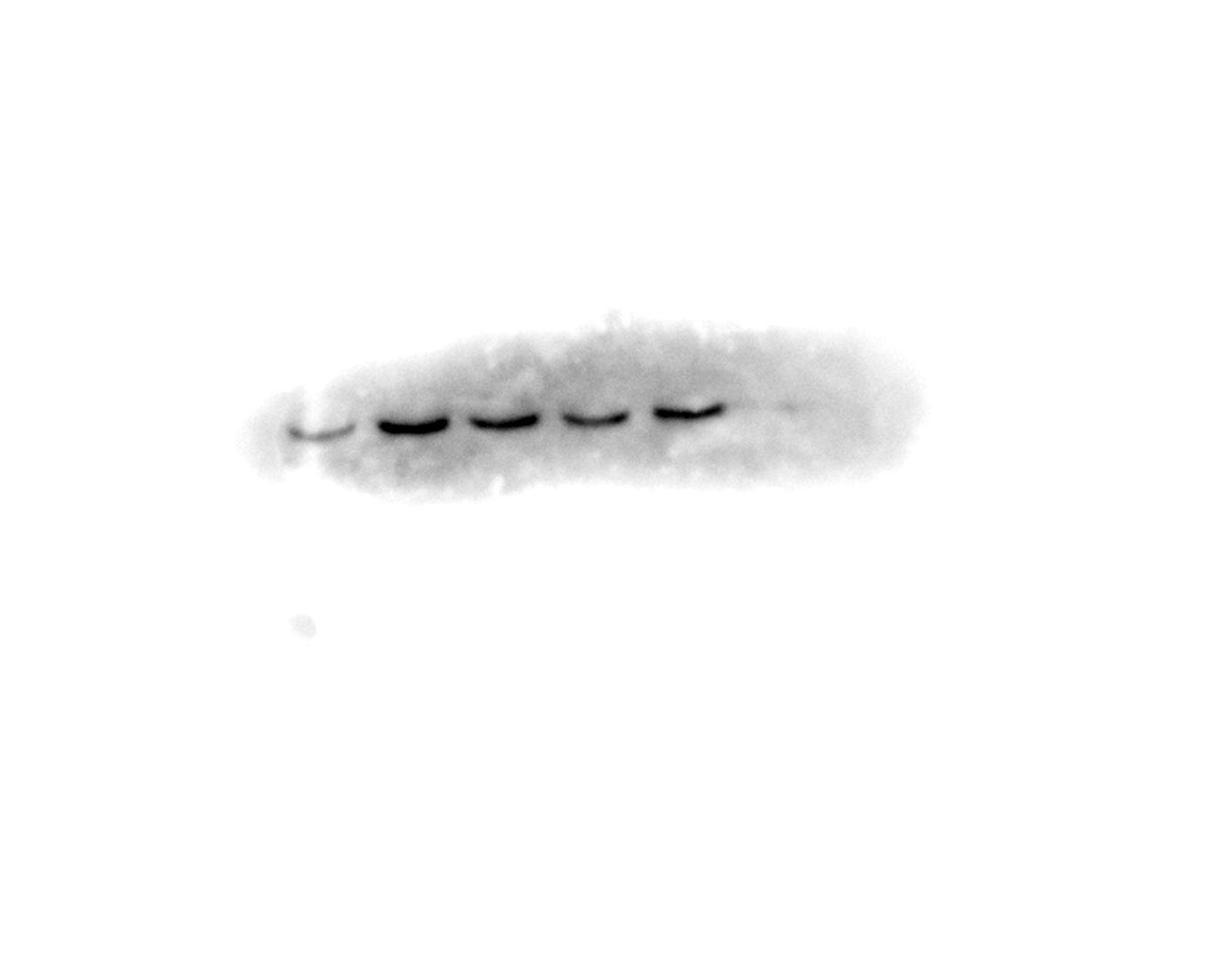

Supplement: Supplementary file 2 [file Data_Sheet_1.zip › Supplement_Data/Experimental Data/WB/retina/VEGFA-1.tif]

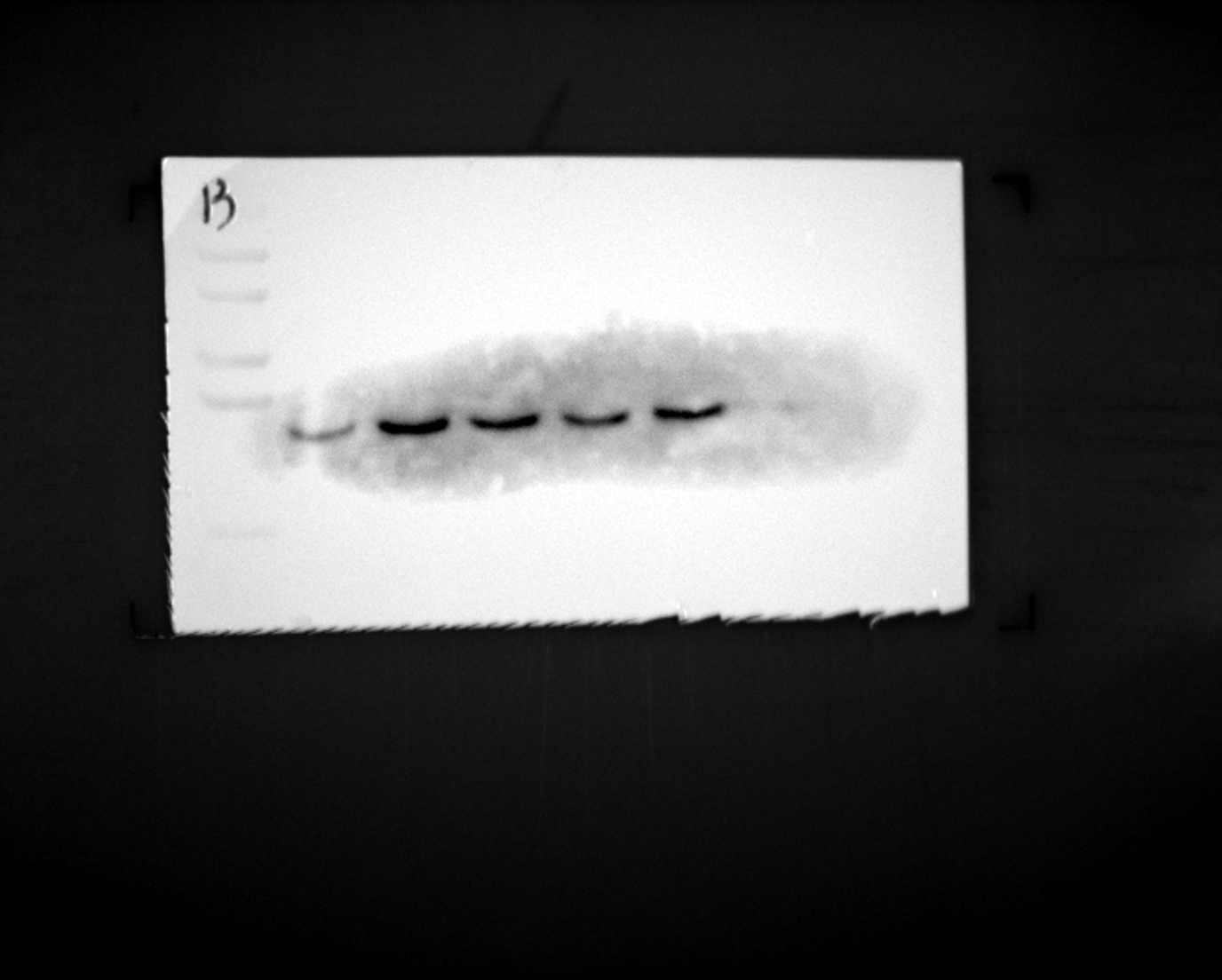

Supplement: Supplementary file 2 [file Data_Sheet_1.zip › Supplement_Data/Experimental Data/WB/retina/VEGFA-1(M).tif]

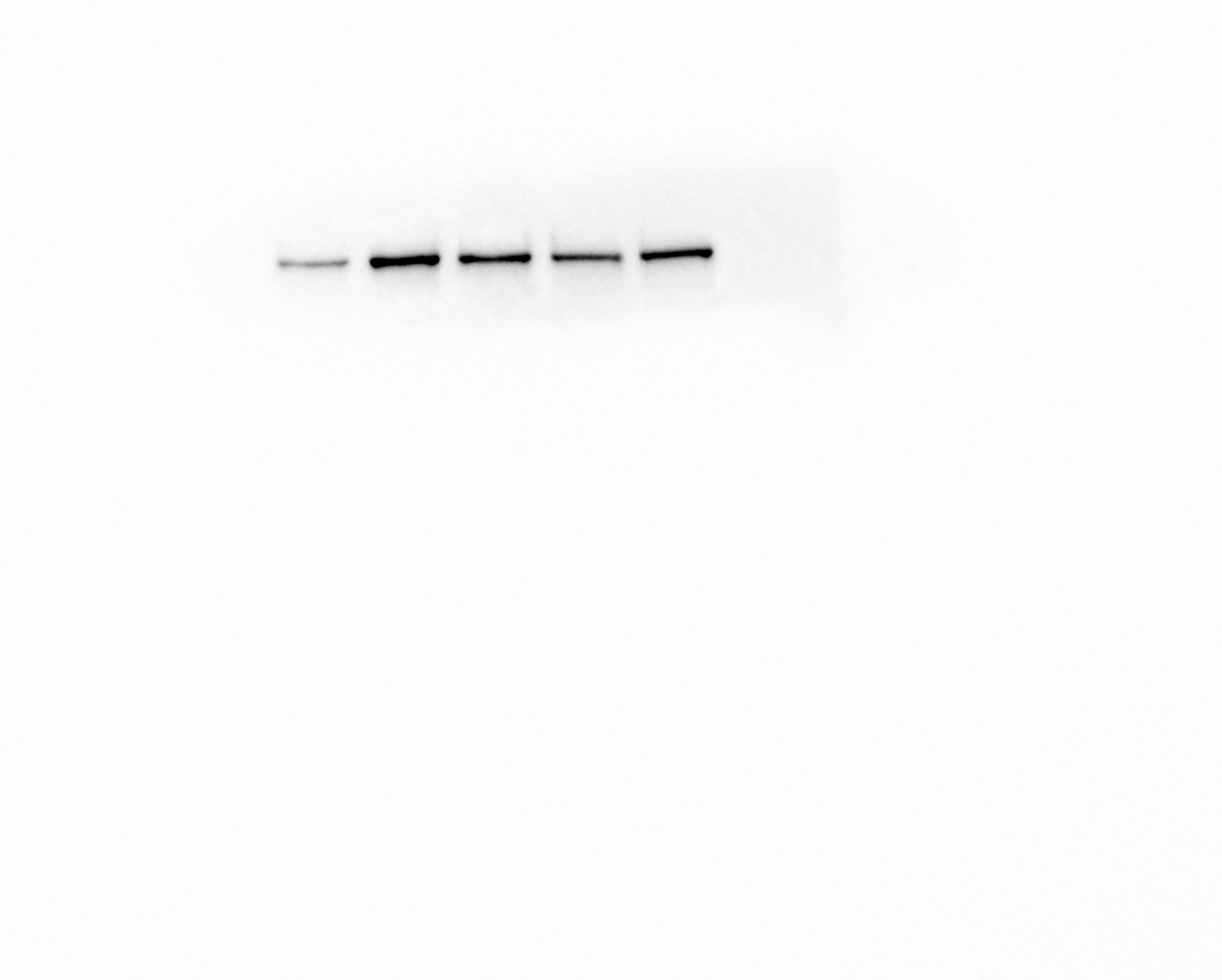

Supplement: Supplementary file 2 [file Data_Sheet_1.zip › Supplement_Data/Experimental Data/WB/retina/VEGFR2-1.tif]

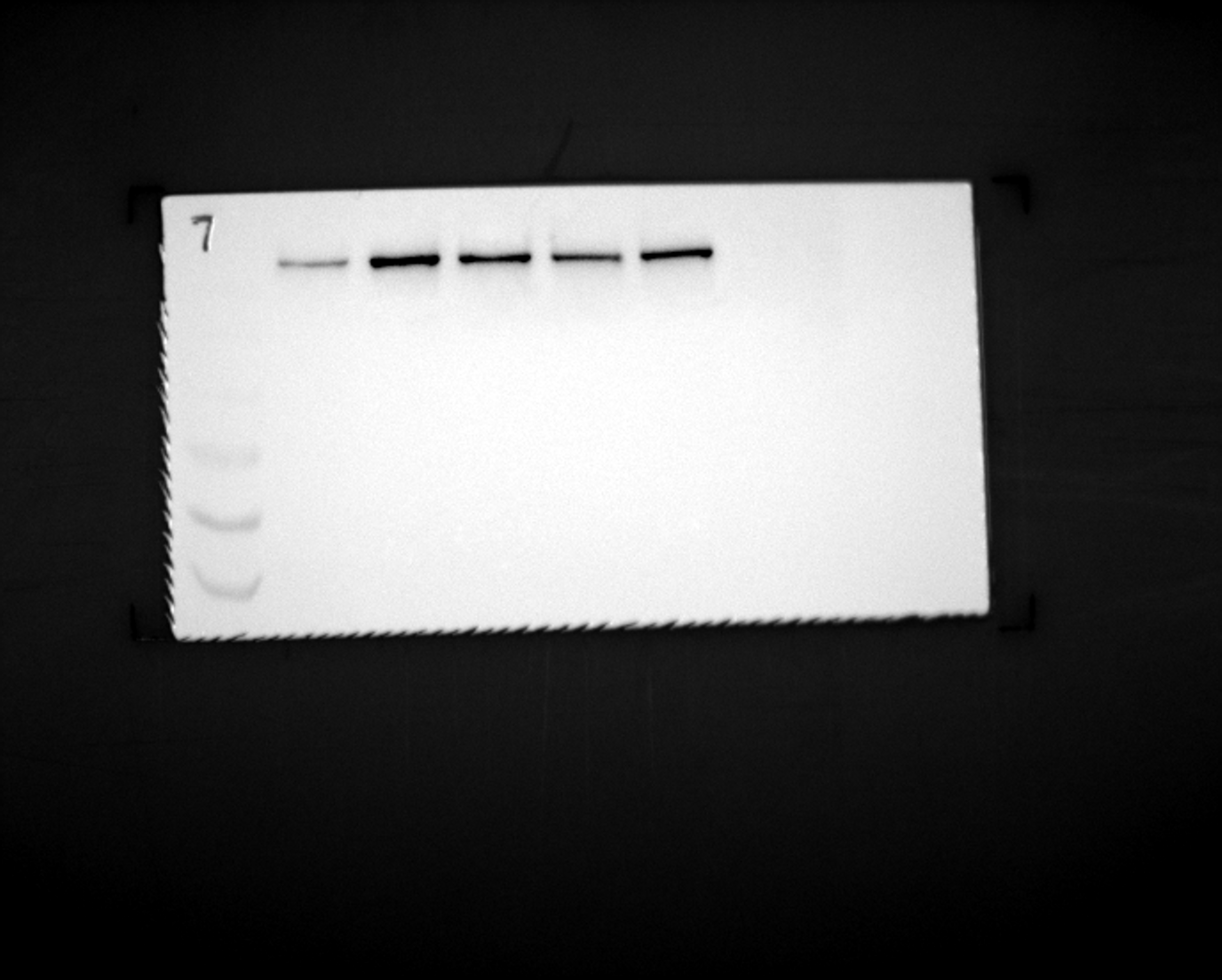

Supplement: Supplementary file 2 [file Data_Sheet_1.zip › Supplement_Data/Experimental Data/WB/retina/VEGFR2-1(M).tif]

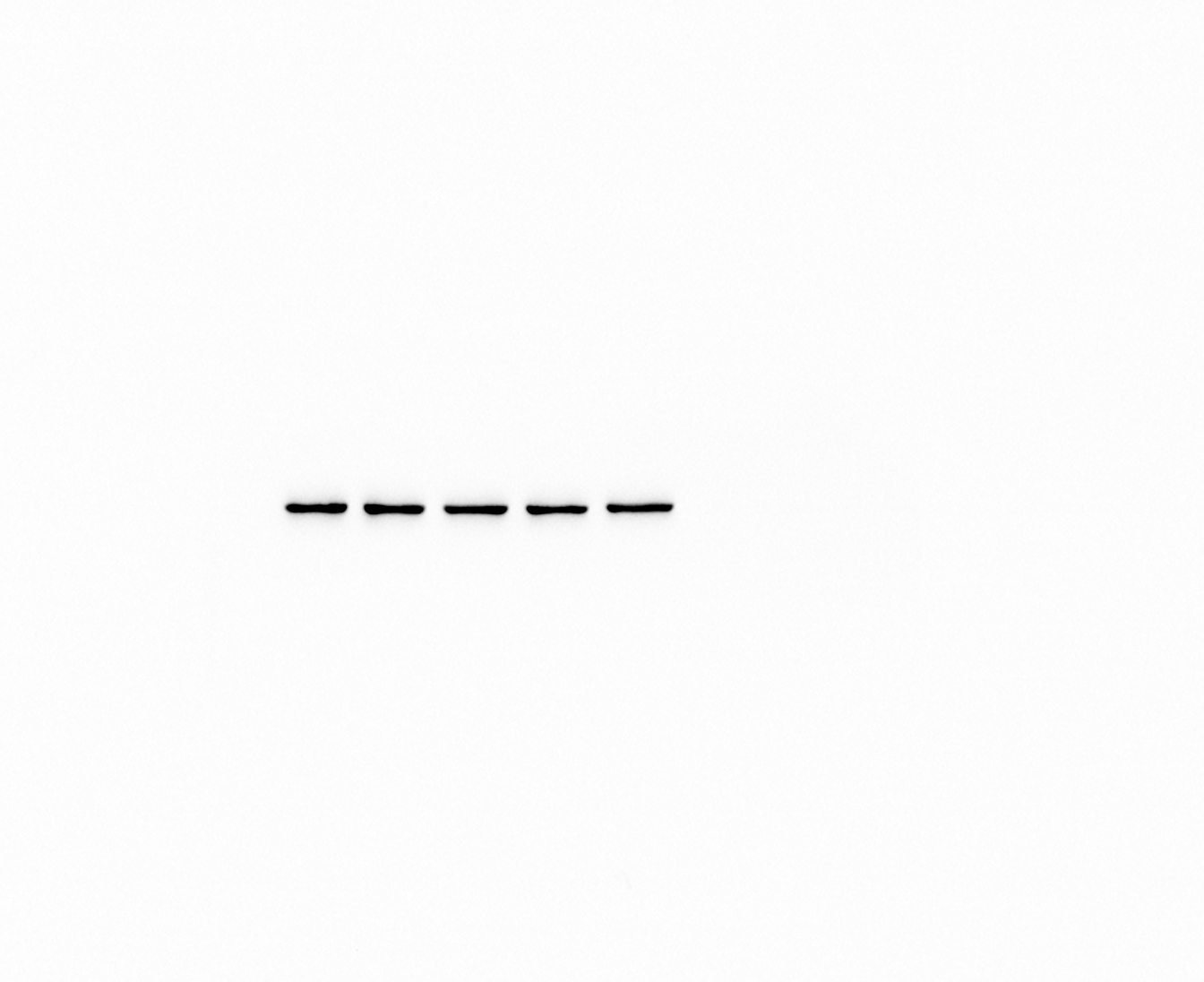

Supplement: Supplementary file 2 [file Data_Sheet_1.zip › Supplement_Data/Experimental Data/WB/retina/β-actin-1.Tif]

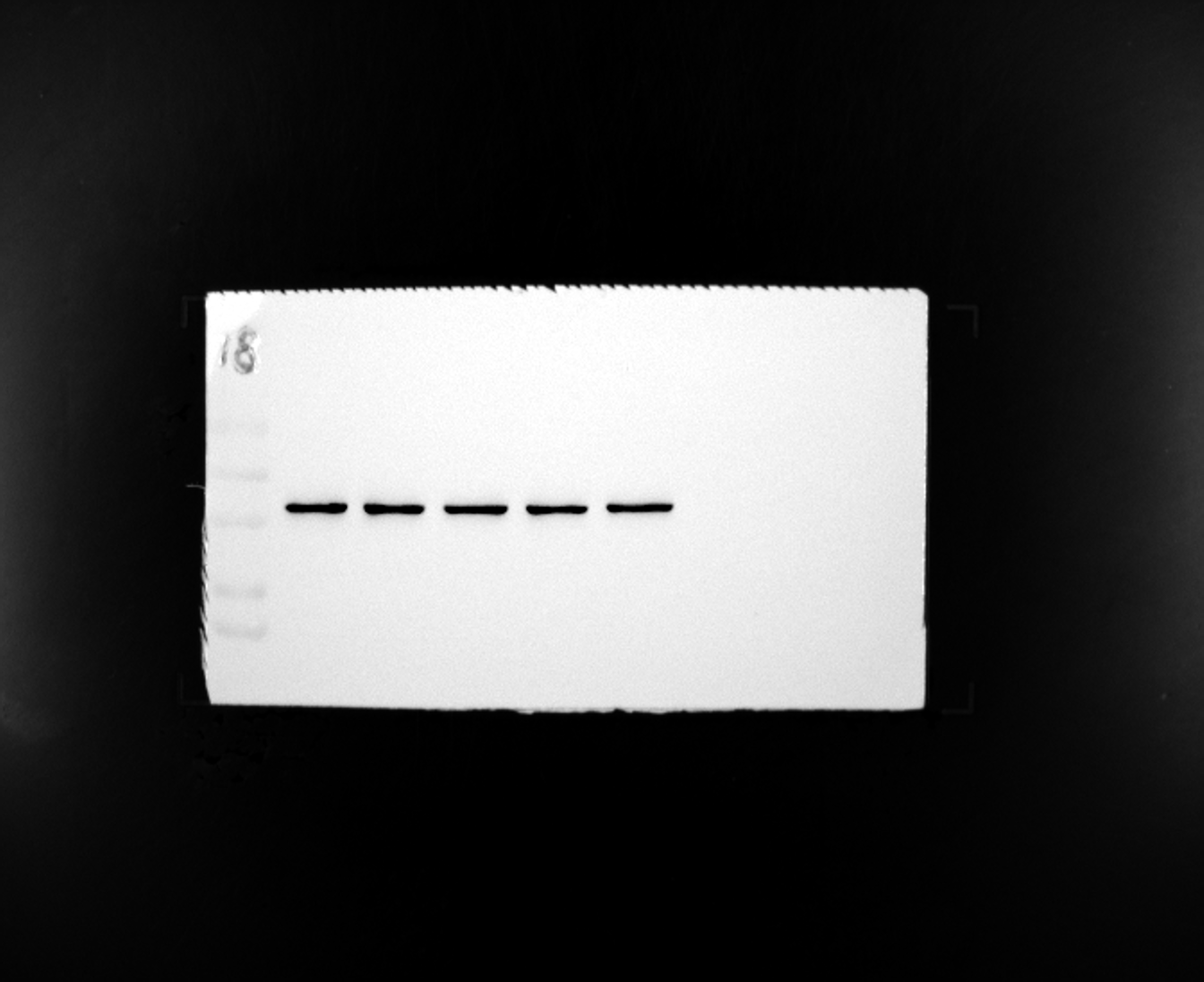

Supplement: Supplementary file 2 [file Data_Sheet_1.zip › Supplement_Data/Experimental Data/WB/retina/β-actin-1(M).Tif]
